# Biogenic Fe_3_O_4_@eggshell Nanocomposite: Synthesis, Physicochemical Properties, and Cr(VI) Removal in Aqueous Media

**DOI:** 10.3390/molecules31183159

**Published:** 2026-09-08

**Authors:** Daniela Camacho-Valencia, Marcelo Rodríguez Valdivia, Gerson Márquez, Fabiana Morales, Jean Juraszek, Christine Devouge-Boyer, Mélanie Mignot, Géraldine Gouhier

**Affiliations:** 1 Doctorado en Ingeniería y Ciencias Ambientales, Universidad Nacional Agraria La Molina, Lima 15024, Peru; 2 Escuela Profesional de Ingeniería de Materiales, Universidad Nacional de San Agustín de Arequipa, Arequipa 04001, Peru; mrodriguezv@unsa.edu.pe; 3 Instituto de Energías Renovables, Universidad Tecnológica del Perú, Arequipa 04001, Peru; 4 Departamento de Física de la Materia Condensada, Centro Atómico Constituyentes, Comisión Nacional de Energía Atómica (CNEA), Buenos Aires B1650, Argentina; fmorales@conicet.gov.ar; 5 Groupe de Physique des Matériaux (GPM), UMR 6634, Université de Rouen Normandie, INSA Rouen Normandie, Centre National de la Recherche Scientifique (CNRS), Normandie Université, F-76000 Rouen, France; jean.juraszek@univ-rouen.fr; 6 Institut de Chimie Analytique et Réactivité Moléculaire en Normandie (CARMeN), UMR 6064, Université de Rouen Normandie, INSA Rouen Normandie, Université de Caen Normandie, École Nationale Supérieure d’Ingénieurs de Caen (ENSICAEN), Centre National de la Recherche Scientifique (CNRS), F-76821 Mont-Saint-Aignan, France; christine.devouge-boyer@insa-rouen.fr (C.D.-B.); melanie.mignot@insa-rouen.fr (M.M.); geraldine.gouhier@univ-rouen.fr (G.G.)

**Keywords:** Fe_3_O_4_@eggshell nanocomposite, green synthesis, hybrid nanomaterials, structure–property relationships, hexavalent chromium removal

## Abstract

A magnetite–eggshell nanocomposite (Fe_3_O_4_@eggshell NC) was synthesized by green coprecipitation and evaluated for Cr(VI) removal from water. *Passiflora ligularis* peel extract, evaluated using a 2^3^ factorial design, served as the biogenic medium, while eggshell waste acted as the support. Characterization included X-ray diffraction (XRD), Fourier-transform infrared spectroscopy (FTIR), scanning and transmission electron microscopy (SEM and TEM), Brunauer–Emmett–Teller (BET) analysis, zeta potential measurements, thermogravimetric analysis (TGA), vibrating sample magnetometry (VSM), and Mössbauer spectroscopy. The NC preserved Fe_3_O_4_ and CaCO_3_ crystalline phases, contained nanoparticles averaging 18 nm, and exhibited oxygenated surface functionalities, a BET surface area of 134.93 m^2^/g, amphoteric behavior, and a predominantly superparamagnetic response suitable for magnetic recovery. Under conditions of pH 4, 180 rpm, 60 min, 0.15 g NC, and 50 mg/L Cr(VI), removal reached 75.98%. Kinetic data followed the pseudo-first-order model, whereas equilibrium data were well described by the Sips isotherm, with an estimated capacity of 50.81 mg/g. At the investigated initial concentration of 50 mg/L, adsorption was exothermic and favored at temperatures up to 308 K, and the material retained moderate reusability during the first three alkaline-regeneration cycles. Overall, Fe_3_O_4_@eggshell NC is a waste-derived, magnetically recoverable adsorbent with favorable Cr(VI) uptake under moderately acidic conditions.

## 1. Introduction

Chromium in aquatic systems occurs mainly in the trivalent and hexavalent states, Cr(III) and Cr(VI), with markedly different mobility and toxicity. Among them, Cr(VI) is of particular concern because it can penetrate biological membranes and induce oxidative stress, leading to DNA damage, genotoxicity and increased cancer risk [1]. Industrial activities such as electroplating, tanning, and pigment manufacturing represent major sources of chromium-bearing effluents, which are often discharged in complex matrices that challenge conventional treatment technologies [2,3,4].

In Latin America, limitations in wastewater treatment infrastructure continue to hinder the effective management of industrial effluents [5]. In countries such as Peru, tanning activities represent a relevant source of chromium-bearing effluents and contaminated sediments [6,7,8]. This context underscores the need for efficient and affordable functional materials that can be produced from locally available resources and applied under complex aqueous conditions for Cr(VI) removal.

Adsorbents derived from renewable or waste-based feedstocks are increasingly explored as alternatives to conventional treatment because they combine high uptake capacity with resource valorization [9]. Additionally, nanostructured sorbents have gained attention because they offer high surface area and reactivity, which can improve treatment efficiency and adsorption selectivity [10]. Magnetite nanoparticles (Fe_3_O_4_ NPs) are particularly attractive because their magnetic properties facilitate solid–liquid separation after treatment, while their surface chemistry supports contaminant uptake [11]. However, they are prone to aggregation and may exhibit limited structural stability.

Green synthesis routes based on plant extracts, microorganisms, or biopolymers have produced Fe_3_O_4_-based materials with demonstrated performance toward dyes, heavy metals and metalloids [12,13,14]. In this context, plant-mediated synthesis is particularly attractive because it can reduce chemical inputs and introduce surface functionalities that contribute to adsorption. Plant extracts used for the green synthesis of Fe_3_O_4_ NPs include *Cordia myxa* [15], *Syzygium aromaticum* [16] and *Azadirachta indica* [17], whereas *Passiflora ligularis* (sweet granadilla) remains unexplored in this context. In Latin America, sweet granadilla is widely cultivated in countries such as Colombia, Brazil, Ecuador, and Peru [18]. *Passiflora* species are rich in bioactive compounds, notably polyphenols and flavonoids, which can promote nanoparticle formation and stabilization [19]. In the case of *P. ligularis*, the peels are a source of carotenoids, polyphenols and pectin, highlighting their potential as a green precursor for nanomaterial synthesis [20]. Moreover, previous studies have shown that these peels have been employed in the eco-friendly synthesis of metal oxide nanoparticles, yielding ultrasmall particles coated with organic phytochemicals [21]. Nevertheless, one critical issue in plant-mediated synthesis is the variability in extract composition, which can directly affect nanoparticle properties and synthesis reproducibility [22]. This limitation highlights the need for better-controlled extract preparation strategies and for clarifying how precursor composition influences the physicochemical features of the resulting material.

To further improve the performance and stability of Fe_3_O_4_-based nanomaterials, surface modification with eggshell waste has been proposed as an effective strategy. Eggshells are a calcium-rich agro-industrial residue that provides a porous mineral scaffold and additional surface functionalities, making them an attractive low-cost support for adsorbent materials. Fe_3_O_4_-eggshell nanocomposites have demonstrated high Cr(VI) removal with good regeneration performance [23,24], and eggshell-derived modifications have also improved the adsorption of antibiotics and herbicides [25,26]. However, despite these advances, studies integrating plant-mediated Fe_3_O_4_ synthesis with eggshell-derived matrices remain scarce. As a result, the structure–property–performance relationship in biogenic Fe_3_O_4_-eggshell systems remains insufficiently understood.

In this context, this study reports the green synthesis of a Fe_3_O_4_@eggshell NC using *Passiflora ligularis* peel extract and eggshell waste as low-cost precursors. Extract preparation was evaluated using a 2^3^ factorial design, while the effects of synthesis pH and eggshell mass on the BET surface area of the nanocomposite were examined using a 2^2^ factorial design. The selected nanocomposite was comprehensively characterized to determine its physicochemical properties. In addition, its Cr(VI) adsorption performance in synthetic aqueous media was evaluated through kinetic, equilibrium, thermodynamic, and regeneration studies. This integrated approach provided a systematic basis for selecting the preparation conditions and enabled interpretation of the selected material’s adsorption behavior in light of its physicochemical properties.

## 2. Results and Discussion

### 2.1. Passiflora ligularis Extract Evaluation

Plant-derived extracts can act as multifunctional media in green syntheses by coupling redox activity with surface conditioning of the forming solids. For *Passiflora ligularis*, flavonoids and phenolic acids (e.g., quercetin, orientin, chlorogenic and caffeic acids) and cyclopassifloside-type terpenoids have been reported as major contributors to reducing power [27]. Consistently, ethanolic extracts of *P. ligularis* peels show a reported ABTS (2,2′-azinobis(3-ethylbenzothiazoline-6-sulfonic acid)) antioxidant capacity of 2.8 ± 0.2 mg Trolox/g along with carotenoids (0.40 ± 0.04 mg/g biomass) and total phenolics (0.13 ± 0.02 mg gallic acid equivalents/g biomass) [20]. These features support their use as a biogenic reaction medium capable of assisting Fe_3_O_4_ formation while introducing organic functionalities that may contribute to nanoparticle stabilization.

The ethanolic extracts of *P. ligularis*, prepared under the conditions defined by the 2^3^ factorial design, showed clear differences in visual appearance, suggesting compositional variability across extraction treatments. Upon addition of DPPH, all extracts induced the expected shift from deep violet to pale yellow, consistent with distinct free-radical scavenging capacities. Quantification at 517 nm confirmed this trend (Table 1): extract E1 achieved the highest inhibition (57.10 ± 0.62%), whereas E4 showed the lowest activity (22.42 ± 0.49%). The CP condition exhibited an intermediate response (48.81 ± 1.53%).

ANOVA revealed significant main effects for all three factors, as well as significant two-factor interactions (A:B, A:C, and B:C; *p* < 0.05), confirming that antioxidant capacity depended on both individual variables and their combined effects, whereas the three-factor interaction (A:B:C) was not significant (*p* > 0.05). The ranked effect estimates further showed that the temperature–time interaction (B:C) was the dominant term overall, whereas among the main effects, temperature (B) had the greatest magnitude, followed by extraction time (C) and ethanol concentration (A). The estimated main effects were negative within the explored domain, indicating that, on average across the factorial design, increasing ethanol concentration, extraction temperature, and extraction time from their low to high levels decreased the inhibition percentage. This behavior may reflect thermal degradation of temperature-sensitive antioxidants, such as flavonoids, at elevated extraction temperatures [28], while higher ethanol concentrations can reduce the solubility of certain polar compounds [29]. Significant curvature was also observed (F = 115.09; *p* < 0.001), indicating a non-linear response within the evaluated range.

Accordingly, E1 was selected for nanocomposite synthesis, as its higher antioxidant response was expected to provide a more controlled biogenic medium for Fe_3_O_4_ formation and interfacial functionalization. Thus, the factorial design contributed to selecting the extract preparation conditions and reducing precursor variability before synthesis. This is relevant because changes in extract composition can affect the surface chemistry and reproducibility of the resulting Fe_3_O_4_@eggshell NC, which in turn may influence its oxygenated surface functionalities and subsequent Cr(VI) adsorption behavior. In this way, the factorial design served as a screening tool for extract selection prior to nanocomposite synthesis.

### 2.2. Green Synthesis of Fe_3_O_4_@eggshell NC

Fe_3_O_4_@eggshell NC was synthesized via an extract-assisted coprecipitation route, where *Passiflora ligularis* extract provided a biogenic medium with reducing potential and surface-stabilizing capacity. Previous reports demonstrated the suitability of other *Passiflora* species for green nanoparticle synthesis [30,31,32]. In the present system, the black coloration observed after the addition of the extract and pH adjustment is consistent with the formation of Fe_3_O_4_ from Fe^3+^ and Fe^2+^ precursors under basic conditions. Antioxidant compounds in the extract may contribute to partial Fe^3+^ reduction and nanoparticle stabilization. Magnetite formation under the present Fe^2+^/Fe^3+^ coprecipitation conditions can be represented by Equation (1):

(1) $$2{Fe}^{3+}+{Fe}^{2+}+8{OH}^{-}+extract\to {Fe}_{3}O_{4}+oxidized\;extract\;compounds+4H_{2}O$$

Pulverized eggshell (CaCO_3_) was incorporated before the final pH adjustment to favor its integration during magnetite formation and promote the development of a hybrid structure. In this system, the carbonate matrix provided a porous substrate for Fe_3_O_4_ deposition and contributed chemically active domains that may facilitate heterogeneous nucleation, reduce particle aggregation, and increase surface accessibility. The resulting assembly likely involved pH-dependent electrostatic effects together with interactions among CaCO_3_ surfaces, Fe–O groups, and extract-derived organic moieties, which may help explain the improved dispersion and accessible surface area of the nanocomposite.

To preserve both the Fe_3_O_4_ phase and organic functional groups, the material was dried at 80 °C without calcination. This low-temperature drying condition helped minimize thermally induced oxidation of Fe_3_O_4_ [33] and preserve the surface chemistry imparted by the green synthesis.

The specific surface area of the resulting nanocomposites was quantified by BET analysis (Table 2). The nanocomposite M3 exhibited the highest BET surface area (155.88 m^2^/g) and was therefore selected for subsequent physicochemical characterization and adsorption experiments. ANOVA with three center points (Table S3) indicated no significant curvature (*p* > 0.05) and identified eggshell mass as the only significant factor influencing BET surface area.

The effect of eggshell mass was negative within the investigated range: increasing the amount from 1.0 to 2.0 g decreased the mean BET surface area from 149.30 to 107.18 m^2^/g. A lower eggshell loading may have provided a more favorable balance between support availability and Fe_3_O_4_ dispersion, whereas increasing the support proportion could have modified particle packing or reduced the accessibility of the porous structure.

Therefore, the eggshell-derived matrix played a significant role in the textural development of the hybrid material, favoring a more accessible porous structure under the selected composition. Since surface area and pore accessibility can influence the exposure of adsorption sites and the diffusion of dissolved species, the 2^2^ factorial design allowed identification of the synthesis condition with the most favorable textural properties for subsequent Cr(VI) adsorption studies.

### 2.3. Physicochemical Properties of Fe_3_O_4_@eggshell NC

#### 2.3.1. FTIR Analysis

**Figure 1:**
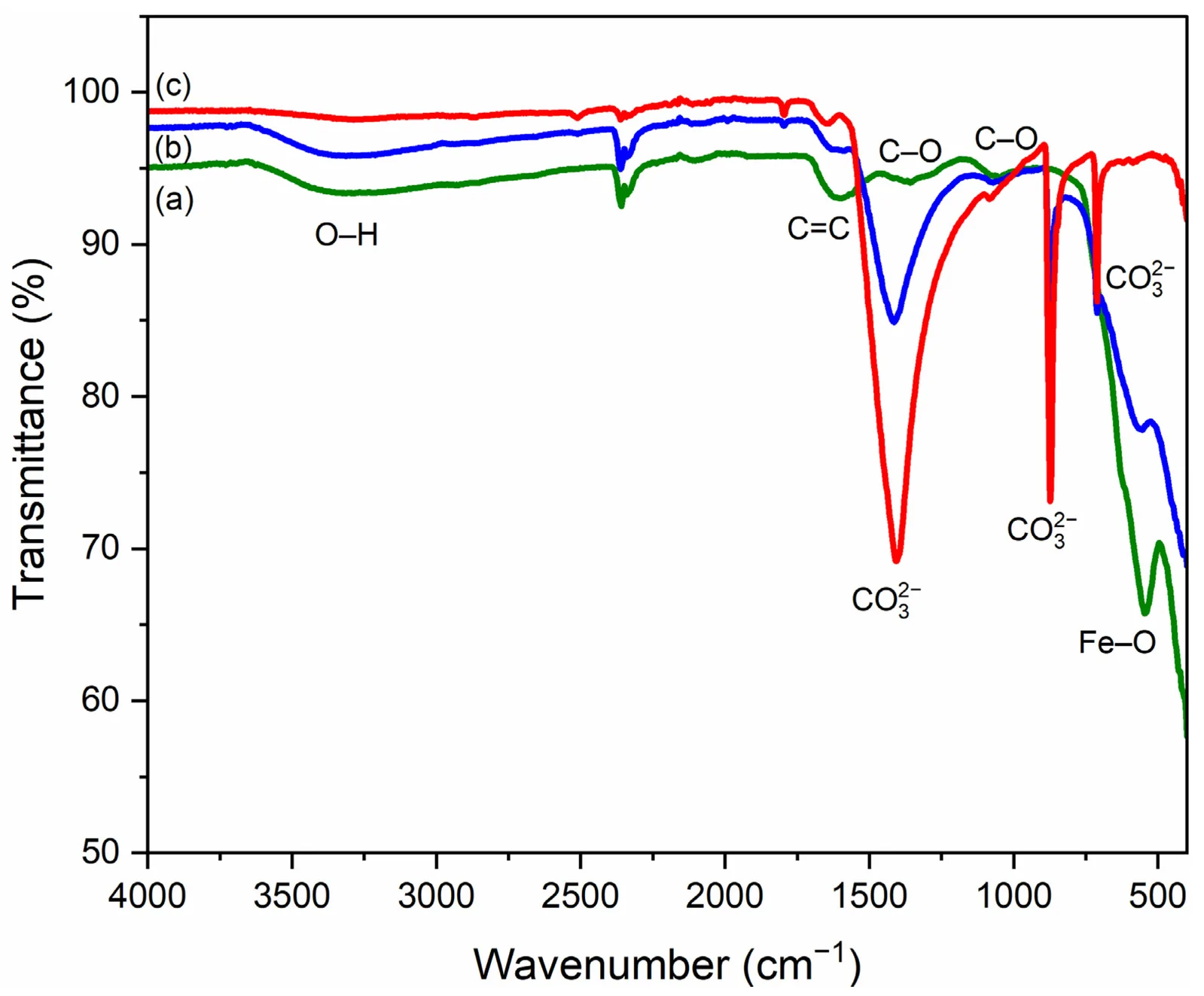
FTIR spectra of (**a**) green-synthesized Fe_3_O_4_ NPs, (**b**) Fe_3_O_4_@eggshell NC and (**c**) eggshell powder.

The FTIR analysis provided insights into the surface chemistry and functional groups present in the synthesized materials, as shown in Figure 1. For the green-synthesized Fe_3_O_4_ NPs (Figure 1a), a broad absorption band centered at approximately 3313 cm^−1^ was attributed to O–H stretching vibrations from surface hydroxyl groups and adsorbed moisture [34]. Weak features observed around 2350 cm^−1^ in the spectra were attributed to atmospheric CO_2_ absorption and were therefore not assigned to the samples. The band at 1601 cm^−1^ can be associated with C=C stretching vibrations of aromatic or conjugated organic moieties, although a contribution from the H–O–H bending vibration of adsorbed water cannot be excluded. Additional bands at approximately 1358 and 1051 cm^−1^ were assigned to oxygenated surface functionalities, particularly C–O and C–O–C vibrations. In addition, the band observed at around 546 cm^−1^ was assigned to Fe–O stretching vibrations of metal ions in tetrahedral sites, which is characteristic of spinel ferrites and supports the formation of magnetite with a cubic spinel structure [35]. Together, these signals supported the presence of extract-derived oxygenated organic functionalities, including hydroxylated and carbonaceous species, which likely remained at the Fe_3_O_4_ surface as a biogenic surface species. Such surface groups are relevant because they can contribute to nanoparticle stabilization during synthesis and provide interfacial sites that may participate in subsequent adsorption processes.

The eggshell spectrum (Figure 1c) exhibited the typical vibrational fingerprint of CaCO_3_ with intense carbonate bands at 1408 cm^−1^ (ν_3_), 874 cm^−1^ (ν_2_) and 712 cm^−1^ (ν_4_) [36]. Additional weaker features at approximately 1795 and 2511 cm^−1^ were also observed, further supporting the predominance of the CaCO_3_ phase.

The Fe_3_O_4_@eggshell NC (Figure 1b) showed the superposition of spectral features from both components. The carbonate bands at 1416, 874, and 712 cm^−1^ indicated the retention of the CaCO_3_ phase after formation of the composite, whereas the Fe–O vibration at 555 cm^−1^ supported the presence of the Fe_3_O_4_ phase. The persistence of the broad O–H band near 3300 cm^−1^ was consistent with hydroxylated surface species in the hybrid material. A band was also observed at approximately 1070 cm^−1^; however, this spectral region may contain overlapping contributions from C–O-containing surface functionalities and carbonate vibrations. The contribution around 1358 cm^−1^ may likewise be partially masked by the intense carbonate band at 1416 cm^−1^.

#### 2.3.2. XRD Analysis

**Figure 2:**
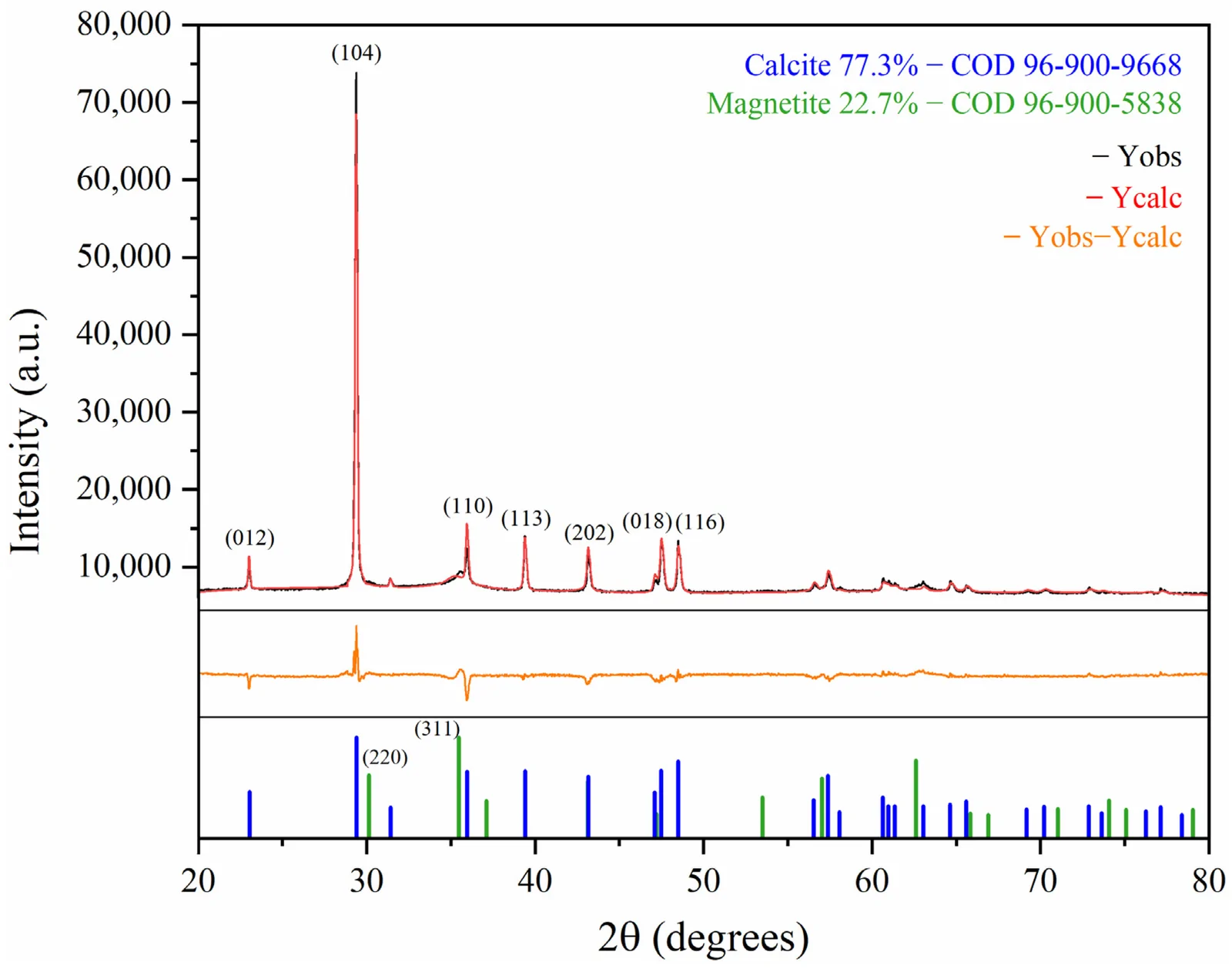
Rietveld refinement of the XRD pattern of the Fe_3_O_4_@eggshell NC. Experimental (Yobs) and calculated (Ycalc) profiles, the difference curve (Yobs−Ycalc), and Bragg reflection positions for calcite (COD 96-900-9668) and magnetite (COD 96-900-5838) are shown. The numbers in parentheses indicate the Miller indices (hkl) assigned to the labeled diffraction peaks.

XRD analysis revealed two dominant crystalline phases corresponding to calcite (77.3%) and magnetite (22.7%), as quantified by Rietveld refinement and reference patterns from the COD database. The main reflections of both crystalline phases were indexed, as shown in Figure 2, with the most intense peaks observed at 2θ = 29.4° and 35.2°, corresponding to the (104) and (311) crystallographic planes of calcite and magnetite, respectively. The diffractogram therefore confirmed the coexistence of CaCO_3_ and Fe_3_O_4_ as the main crystalline phases in the nanocomposite.

No secondary iron oxide phases, such as hematite or iron oxyhydroxides, were detected, indicating that the synthesis and low-temperature drying conditions preserved the magnetite phase. The comparatively broader magnetite reflections are consistent with the presence of nanocrystalline domains. The crystallite size of magnetite, estimated from the (311) reflection using the Scherrer equation, was approximately 5.17 nm.

#### 2.3.3. Morphological and Elemental Evaluation

The SEM images of eggshell powder (Figure S1a) revealed a heterogeneous morphology composed of fractured, angular and irregularly shaped particles. The surfaces displayed a rugged texture with granular domains and micro-fractures, evidencing a non-uniform architecture that may increase surface accessibility and provide multiple anchoring sites for nanoparticle deposition and interfacial interactions. Eggshell is considered a spongy biomaterial with layered domains and microcavities [23,37], which supports its suitability as a natural matrix for surface modification and composite formation. The EDS spectrum (Figure S1b) confirmed calcium and oxygen as the predominant elements, with trace magnesium levels, consistent with CaCO_3_ as the major constituent of the eggshell substrate.

The green-synthesized Fe_3_O_4_ NPs formed compact micrometric agglomerates with irregular contours and no clearly isolated primary particles (Figure S2a), a morphology commonly associated with magnetic dipolar interactions and the high surface energy of nanophases [38]. EDS analysis (Figure S2b) showed intense Fe Kα/Kβ signals together with O, consistent with an iron oxide phase and in agreement with previous reports on green-synthesized magnetite [39,40]. Minor Na and K signals likely originated from residual synthesis components and extract-derived species, respectively, whereas the C signal is consistent with extract-derived organic species associated with nanoparticle capping. This agglomerated morphology supports the use of a solid substrate such as eggshell to improve phase distribution and limit particle clustering.

**Figure 3:**
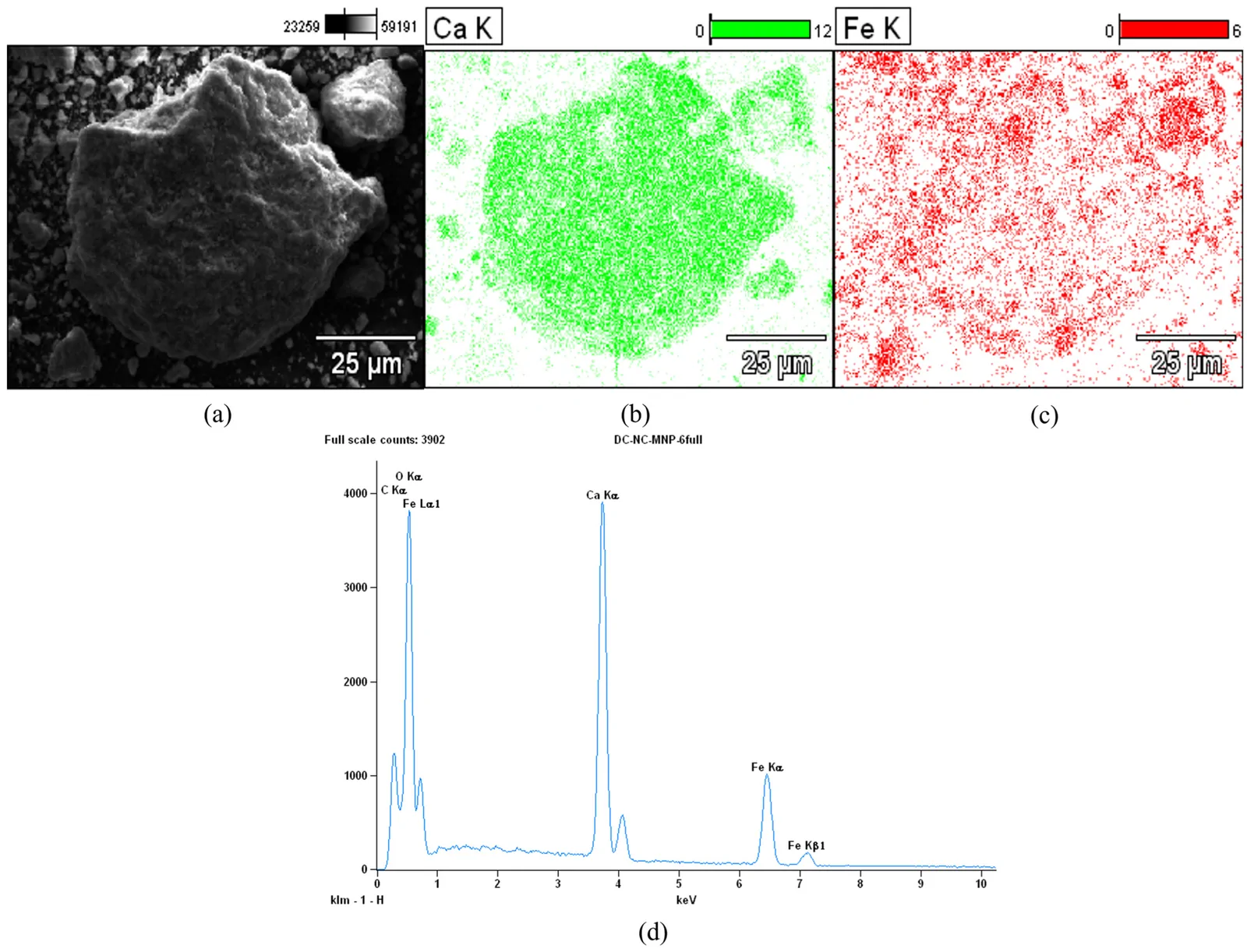
(**a**) SEM image, (**b**) elemental maps of Ca K, (**c**) Fe K and (**d**) EDS spectrum of the Fe_3_O_4_@eggshell NC.

For the Fe_3_O_4_@eggshell NC, SEM revealed grains comparable in size to the pristine eggshell, yet with a finer surface texture decorated by nanoscale domains (Figure 3a). Elemental mapping evidenced Ca-rich regions attributable to the carbonate matrix and Fe-enriched domains preferentially distributed near the external surface (Figure 3b,c), suggesting that Fe_3_O_4_ NPs are mainly deposited on the support surface rather than being uniformly embedded throughout the matrix [26,41,42]. The EDS spectrum further confirmed the concurrent presence of Ca, O, and Fe in the nanocomposite (Figure 3d), while the semi-quantitative EDS results (Table S4) highlighted the compositional contrast among eggshell, Fe_3_O_4_ NPs, and Fe_3_O_4_@eggshell NC. Together, these observations are consistent with a hybrid material in which the eggshell-derived CaCO_3_ phase preserved its rough mineral morphology and acted as the supporting matrix, while the Fe_3_O_4_ component remained associated with its surface.

**Figure 4:**
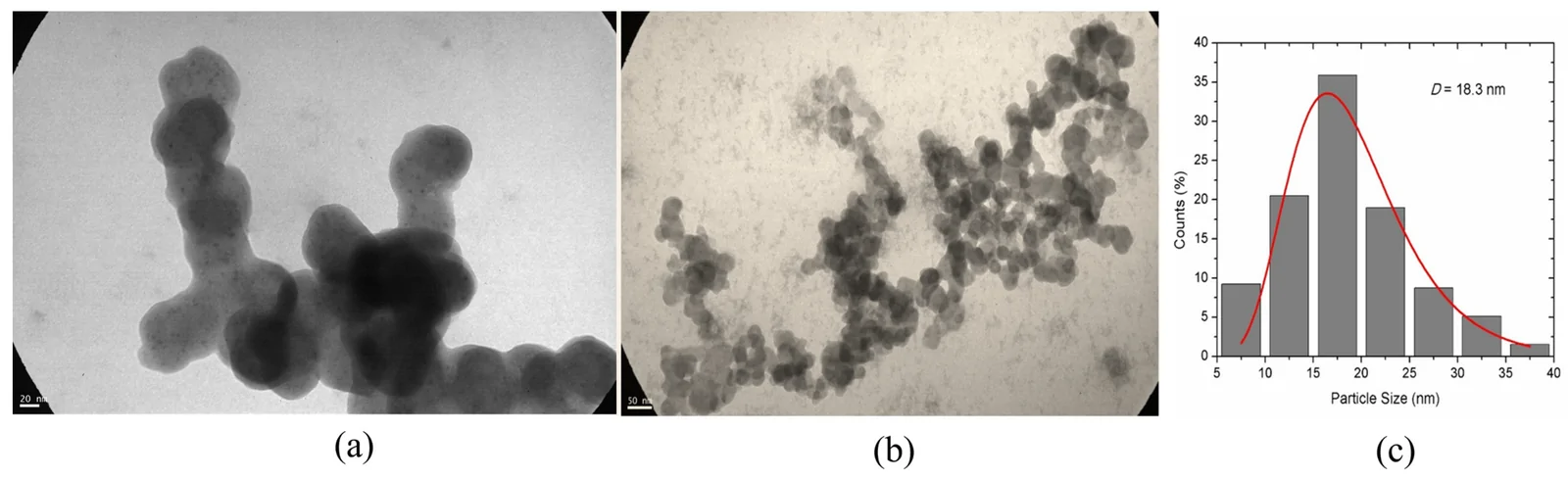
(**a**,**b**) TEM images of Fe_3_O_4_@eggshell NC showing non uniformly distributed nanoparticles and (**c**) particle size distribution histogram. The red line in panel (**c**) represents the lognormal fit to the particle size distribution.

TEM micrographs of the Fe_3_O_4_@eggshell NC are presented in Figure 4a,b. In Figure 4a, quasi-spherical, electron-dense nanoparticles consistent with the Fe_3_O_4_ phase are observed over a lower-contrast region that may correspond to the CaCO_3_-rich matrix, supporting the formation of a hybrid material. In some particles, a faint contrast gradient from core to edge can be observed, which may be associated with interfacial layering or with a surface coating derived from carbonate species. However, conventional TEM does not allow definitive confirmation of a core–shell configuration. Further HRTEM and/or STEM analyses would help to assess the Fe_3_O_4_/CaCO_3_ interfacial organization more rigorously. Similar contrast separation between an electron-dense magnetic phase and a lighter Ca-based component has been reported for related composites [42], which is consistent with a surface-supported Fe_3_O_4_ dispersion in the present system.

Figure 4b displays predominantly spherical nanoparticles organized into chain-like and branched assemblies, a morphology commonly associated with magnetic dipolar interactions among magnetite nanodomains. The particle size histogram (Figure 4c) indicates diameters ranging from 7 to 38 nm, with an average size of 18.3 nm. The larger mean particle size observed by TEM compared with the crystallite size estimated by XRD (5.17 nm) suggests that the particles may comprise multiple crystalline domains. This size range is consistent with nanoscale Fe_3_O_4_ domains and supports the magnetic behavior discussed later, since particles of this scale are commonly associated with predominantly superparamagnetic response at room temperature.

#### 2.3.4. Surface Study

Nitrogen adsorption–desorption measurements revealed clear differences in the textural properties of the analyzed materials. The unmodified eggshell exhibited a very low BET surface area (1.56 m^2^/g), a pore volume of 0.00368 cm^3^/g, and an average pore diameter of 10.28 nm, in agreement with values reported for uncalcined eggshells [43]. The slightly smaller pore diameter observed in the present work likely relates to the fine particle size used (≤75 µm), which can increase packing density and reduce the contribution of larger interparticle voids.

**Figure 5:**
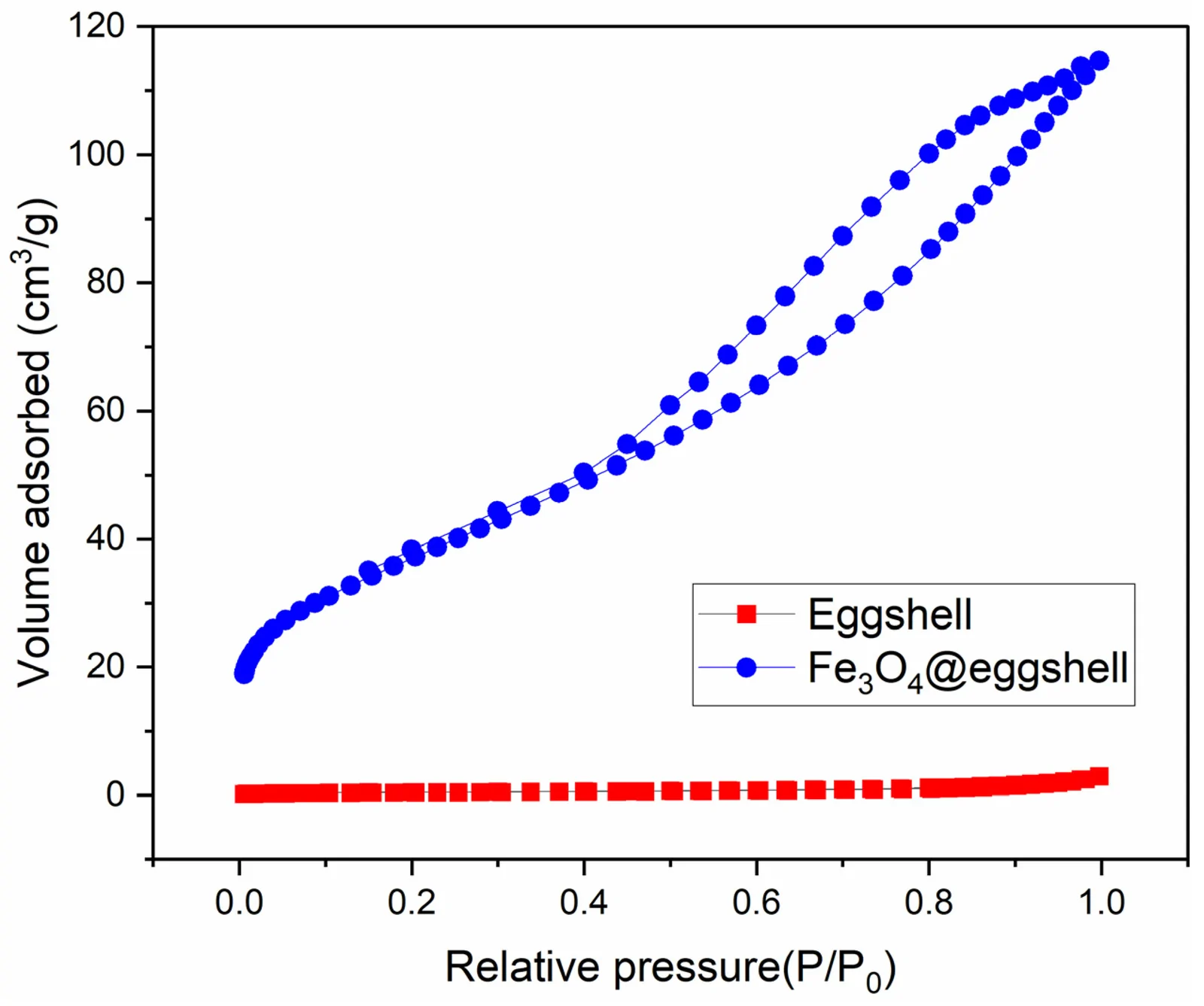
N_2_ adsorption–desorption isotherms of the unmodified eggshell and the Fe_3_O_4_@eggshell NC.

As shown in Figure 5, both the eggshell and the Fe_3_O_4_@eggshell NC displayed type IV isotherms with H3 hysteresis loops, commonly associated with mesoporous solids containing slit-like pores and/or aggregates of plate-like particles. The Fe_3_O_4_@eggshell NC exhibited a markedly higher nitrogen uptake and a more pronounced hysteresis loop, indicating a substantial modification of the textural properties after incorporation of the magnetic phase. This material, prepared from a separate batch under the selected M3 synthesis conditions, showed a BET surface area of 134.93 m^2^/g, a pore volume of 0.17340 cm^3^/g and an average pore diameter of 4.97 nm. Compared with pristine eggshell, this large increase in surface area and pore volume, together with the preservation of mesoporosity, indicates the development of a much more accessible interfacial structure. Such textural changes are relevant to material functionality because they can favor contact between dissolved species and available adsorption sites during subsequent Cr(VI) uptake experiments. Similar textural evolution has been reported for Fe_3_O_4_-based hybrids supported on mineral or bio-derived matrices [44]. Compared with chemically synthesized Fe_3_O_4_/eggshell systems reported at higher eggshell-to-magnetite ratios [24], the present textural data suggest that porosity is sensitive to both composition and synthesis route.

#### 2.3.5. Surface Charge Behavior

Zeta potential measurements were used to determine the pH-dependent surface charge of the green Fe_3_O_4_ NPs and the Fe_3_O_4_@eggshell NC (Figure S3). Both materials exhibited the typical amphoteric behavior of Fe_3_O_4_, with positive potentials under acidic conditions and progressively negative values as pH increased, consistent with protonation/deprotonation of surface hydroxyl groups. For the green Fe_3_O_4_ NPs, the isoelectric point (IEP) occurred at pH 6.06, with a zeta potential of −17.2 mV at pH 7, suggesting a predominantly negative surface charge with limited electrostatic stabilization against aggregation. Similar magnitudes have been reported for plant-derived magnetite systems, such as those synthesized from marigold extracts [45], *Hagenia abyssinica* precursors [46], and *Rhus coriaria* [47], where oxygenated phytochemicals modulate surface charge and interparticle interactions.

In contrast, the Fe_3_O_4_@eggshell NC exhibited a slightly lower IEP (pH 5.57) and a less negative potential at neutral pH (−8.8 mV), indicating a shift in surface acidity after composite formation. Comparable behavior was observed by Elshimy et al. [36] for Fe_3_O_4_/eggshell alginate composites where carboxylates increased the negative surface charge at pH 7. Conversely, Ajab et al. [23] reported a higher IEP (around pH 8) for chemically synthesized Fe_3_O_4_ and eggshell systems without an organic capping layer. Differences in synthesis route, surface chemistry, and electrolyte conditions can lead to variations in the apparent IEP, particularly for carbonate-containing supports where solution speciation may contribute to the measured potential. In the present hybrid, the lower IEP and less negative charge at neutral pH are consistent with the combined contribution of the carbonate matrix and extract-derived oxygenated moieties to the interfacial charge response.

From a functional perspective, the positive surface charge developed under acidic conditions is relevant because it favors electrostatic attraction of anionic Cr(VI) species. As pH increases and the surface becomes progressively negative, electrostatic attraction is expected to diminish. Therefore, zeta potential results support the pH-dependent adsorption behavior of the nanocomposite and suggest that electrostatic effects contribute to Cr(VI) uptake.

#### 2.3.6. TGA

The thermal behavior of the eggshell, green-synthesized Fe_3_O_4_ NPs and Fe_3_O_4_@eggshell NC is presented in Figure S4. The eggshell exhibited the largest overall mass loss (approximately 46%), with an initial contribution between 33 and 353 °C, associated with moisture release and decomposition of volatile and organic fractions, followed by the characteristic high-temperature decarbonation of CaCO_3_ centered at 814 °C [48]. Avian-derived membranes rich in proteinaceous functional groups have been reported to undergo early thermal degradation while retaining chemically active sites relevant to chromium adsorption [49].

In contrast, the green-synthesized Fe_3_O_4_ NPs showed higher thermal stability, with a total mass loss of 15.6%. The initial event at 89 °C was attributed to desorption of surface water, whereas the contributions between 283 and 381 °C were associated with the progressive removal of surface hydroxyls and extract-derived organic moieties. Additional features between 604 and 758 °C are consistent with thermally induced changes in the inorganic fraction [50].

As expected for a hybrid system, the Fe_3_O_4_@eggshell NC presented an intermediate behavior with a total loss of 32.3%. The initial event below 100 °C corresponded to water removal, while the mass losses in the 200–400 °C range were attributed to decomposition of extract-derived organic species, supporting the persistence of biogenic surface functionalities after synthesis. The major high-temperature event centered around 760 °C was attributed to CaCO_3_ decarbonation, while the additional contribution above 800 °C may also involve high-temperature transformations of the iron oxide phase. The remaining mass fraction (67.7%) reflected the combined contribution of the calcium-containing inorganic residue and the iron oxide phase, indicating that the hybrid retained a substantial inorganic framework after thermal treatment. The TGA profile supports the coexistence of organic surface species with thermally stable mineral and iron-containing components in the nanocomposite.

#### 2.3.7. Magnetization Evaluation

**Figure 6:**
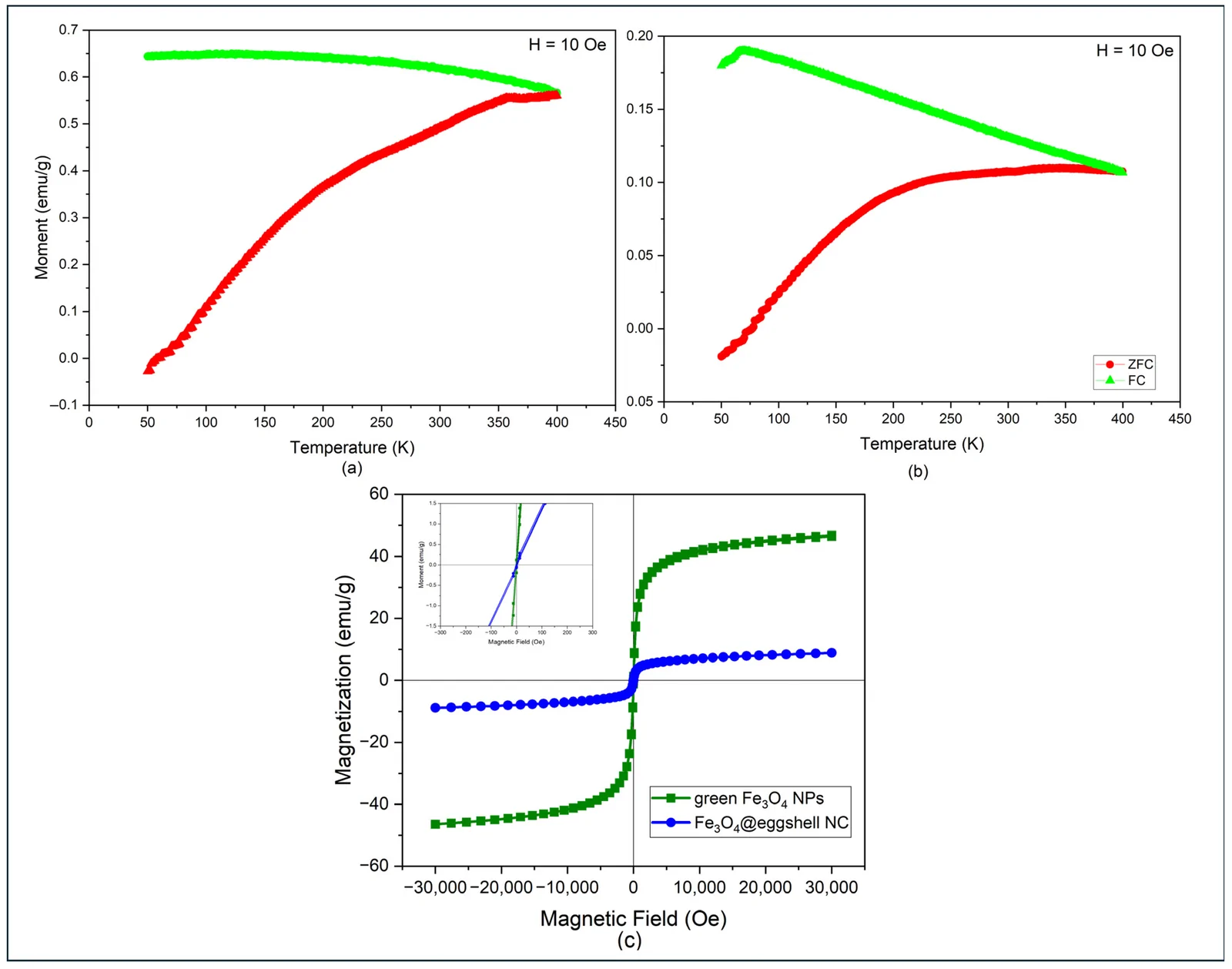
(**a**) ZFC and FC magnetization curves of green-synthesized Fe_3_O_4_ NPs measured under an applied field of 10 Oe. (**b**) ZFC and FC magnetization curves of Fe_3_O_4_@eggshell NC measured under an applied field of 10 Oe. (**c**) Room-temperature hysteresis curve of the green-synthesized Fe_3_O_4_ NPs and the Fe_3_O_4_@eggshell NC. The inset in (**c**) shows an enlarged view of the low-field region.

The temperature dependence of magnetization was evaluated under zero-field-cooled (ZFC) and field-cooled (FC) conditions between 50 and 400 K, as shown in Figure 6a,b. Both the green-synthesized Fe_3_O_4_ NPs and the Fe_3_O_4_@eggshell NC exhibited the typical response of nanoparticle systems with a broad distribution of magnetic energy barriers. For the Fe_3_O_4_ NPs (Figure 6a), the ZFC curve increases with temperature and displays a slight change in slope near 350 K, consistent with a wide blocking-temperature distribution [51]. The FC curve remains above the ZFC curve over the full temperature range up to 400 K, indicating that a fraction of the largest magnetic particles remain magnetically blocked within the measurement window, whereas smaller particles progressively approach a thermally activated, superparamagnetic regime.

Dispersing a magnetic phase within a diamagnetic matrix generally leads to a reduced magnetization. Accordingly, the Fe_3_O_4_@eggshell NC (Figure 6b) shows a qualitatively similar ZFC/FC behavior but with markedly lower magnetization, attributable to the CaCO_3_ matrix. The ZFC curve reaches a plateau near 250 K, which may reflect reduced interparticle magnetic interactions after incorporation of Fe_3_O_4_ domains into the eggshell-derived matrix. The persistence of ZFC–FC separation up to 400 K likewise supports the presence of a high-temperature blocked fraction associated with the largest domains. In addition, the FC curve shows a low-temperature feature around 70 K, which may be associated with magnetic disorder or spin reorientation effects reported for spinel-type systems [52].

Room-temperature magnetization curves recorded as a function of applied field for both the green-synthesized Fe_3_O_4_ NPs and the Fe_3_O_4_@eggshell NC (Figure 6c) show narrow and symmetric hysteresis loops, with very low coercivity and remanence as highlighted in the enlarged low-field region shown in the inset. This behavior indicates that the magnetic response at 300 K was predominantly superparamagnetic, with a minor blocked contribution attributable to the largest particles. The magnetic parameters extracted from the loops are summarized in Table S5.

The saturation magnetization of the green-synthesized Fe_3_O_4_ NPs (M_S_ = 46.4 emu/g) remained below the bulk value (92 emu/g at 20 °C) [53], which may be attributed to surface spin disorder and to the presence of extract-derived organic species at the nanoparticle surface. This value falls within the range commonly reported for biogenic Fe_3_O_4_ NPs, for which M_S_ depends strongly on particle size and surface chemistry [54,55,56]. After composite formation, M_S_ decreased to 8.7 emu/g, reflecting the contribution of the non-magnetic CaCO_3_ phase. Despite this decrease, the NC retains a predominantly superparamagnetic behavior, with very low coercivity and remanence, indicating good responsiveness to an applied magnetic field. Similar M_S_ values have been reported for eggshell and CaO-based Fe_3_O_4_ composites [42,44,57]. This feature is functionally relevant because it supports rapid separation of the adsorbent under an external magnetic field, which is advantageous for recovery and reuse in aqueous treatment applications.

**Figure 7:**
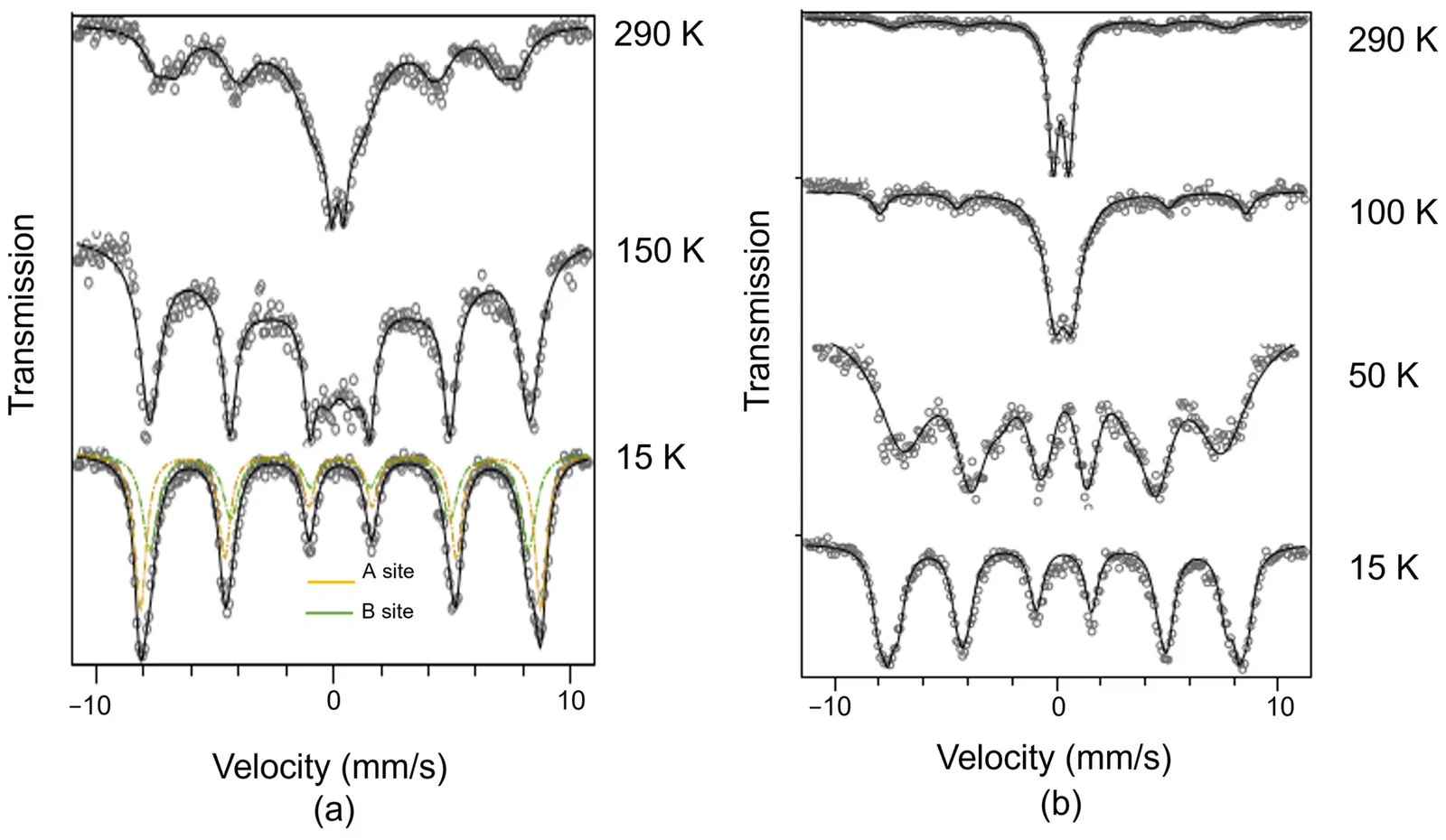
^57^Fe Mössbauer spectra of (**a**) Fe_3_O_4_ NPs at 290 K, 150 K and 15 K and (**b**) Fe_3_O_4_@eggshell NC at 290 K, 100 K, 50 K, and 15 K.

Figure 7 presents the ^57^Fe Mössbauer spectra of the green-synthesized Fe_3_O_4_ NPs (Figure 7a) and the Fe_3_O_4_@eggshell NC (Figure 7b) recorded at different temperatures. At 290 K, both materials exhibit a central doublet, consistent with superparamagnetic relaxation at room temperature, indicating that the magnetic moments fluctuate rapidly on the Mössbauer timescale. Upon cooling down to 15 K, magnetically split sextets progressively emerge and sharpen, evidencing the transition from a thermally activated superparamagnetic regime to a magnetically blocked state, as expected for nanoscale Fe_3_O_4_.

The spectral components of the Fe_3_O_4_ NP spectrum at 15 K were consistent with the two crystallographic iron environments of magnetite, namely Fe^3+^ in tetrahedral (A) sites (45%) and mixed-valence Fe^2+^/Fe^3+^ in octahedral (B) sites (55%), supporting the ferrimagnetic nature of the magnetic phase. At the same temperature, the Mössbauer spectrum of Fe_3_O_4_@eggshell NC exhibits broader lines, suggesting that the CaCO_3_ matrix and the Fe_3_O_4_–CaCO_3_ interface influenced the local Fe environment. Comparison of the thermal evolution of both sample spectra suggests that the nanocomposite exhibits a lower blocking temperature than the Fe_3_O_4_ NPs. Mössbauer features are consistent with the magnetization results (Figure 6), supporting the coexistence of blocked and rapidly relaxing magnetic fractions over the investigated temperature range. Thus, Mössbauer spectroscopy supports the assignment of magnetite as the iron oxide phase and provides local structural evidence that incorporation into the eggshell-derived matrix modifies the Fe environment and magnetic relaxation behavior.

Overall, the combined structural, textural, magnetic, and electrokinetic results confirm the successful synthesis of a functional Fe_3_O_4_@eggshell NC. These properties support its evaluation as a magnetically recoverable adsorbent for Cr(VI) removal from aqueous media.

### 2.4. Operational Screening for Batch Cr(VI) Adsorption

Batch adsorption performance was evaluated from the residual Cr(VI) concentration measured after each experiment, from which removal efficiency and adsorption capacity were determined. To define the operating conditions for subsequent assays, solution pH, agitation speed, contact time, adsorbent dosage, and initial Cr(VI) concentration were examined. The screening results are discussed according to their influence on interfacial control, hydrodynamic behavior, equilibration, and the balance between available adsorption sites and contaminant loading.

#### 2.4.1. Interfacial Chemistry Under Acidic Conditions

**Figure 8:**
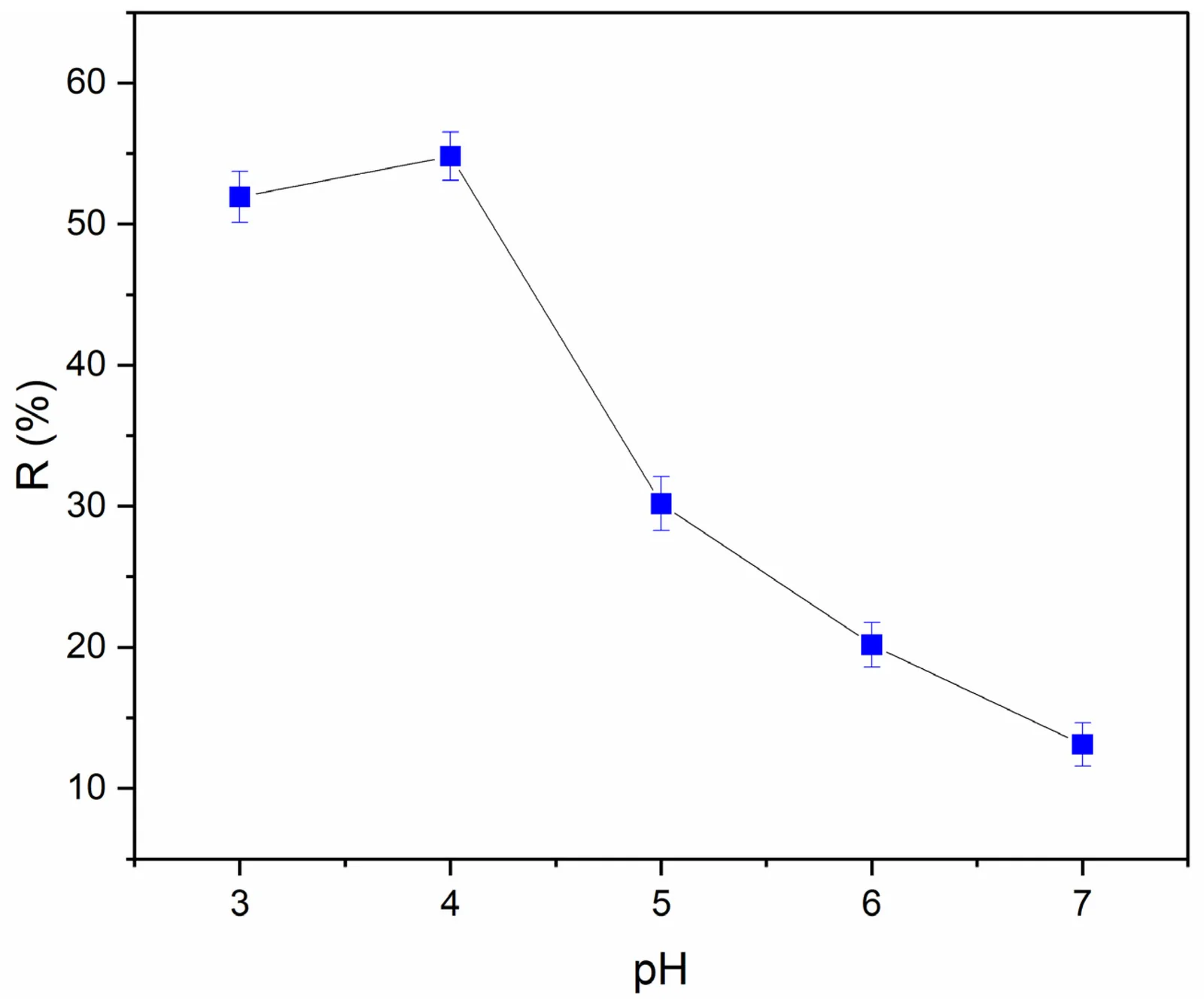
Effect of solution pH on Cr(VI) removal by Fe_3_O_4_@eggshell NC under batch conditions. Blue squares represent mean values, and error bars indicate the standard deviation of three measurements (n = 3).

The adsorption response of Fe_3_O_4_@eggshell NC was strongly affected by solution pH as shown in Figure 8. Across the investigated range, Cr(VI) removal was favored under acidic conditions and declined markedly as pH increased. The highest removal efficiency was obtained at pH 4 (54.82%), followed by pH 3 (51.94%), whereas performance decreased progressively toward neutral conditions, reaching only 13.11% at pH 7. Because adsorption became severely limited in this region, alkaline conditions were not explored further. Based on these results, pH 4 was selected for the subsequent adsorption experiments.

This behavior is consistent with the interfacial chemistry of the system. Under acidic conditions, Cr(VI) is predominantly present as ${HCrO}_{4}^{-}$ and ${Cr}_{2}O_{7}^{2-}$ [49], while protonation of surface sites favors electrostatic attraction between the adsorbent and these anionic species [16]. As pH increases, this favorable electrostatic environment weakens while competition from OH^−^ may also contribute to the lower chromium uptake [44]. This interpretation is supported by the surface-charge data: the nanocomposite remained positively charged at pH 4, and its isoelectric point was 5.57, indicating that adsorption should be favored below this value.

Similar pH optima have been reported for magnetite-containing adsorbents and related biocomposites [44,58,59]. More generally, the preference for acidic conditions agrees with previous studies identifying the pH 2–4 range as the most suitable interval for the adsorption of anionic Cr(VI) species [24].

#### 2.4.2. Hydrodynamic Regime and Equilibration Behavior

**Figure 9:**
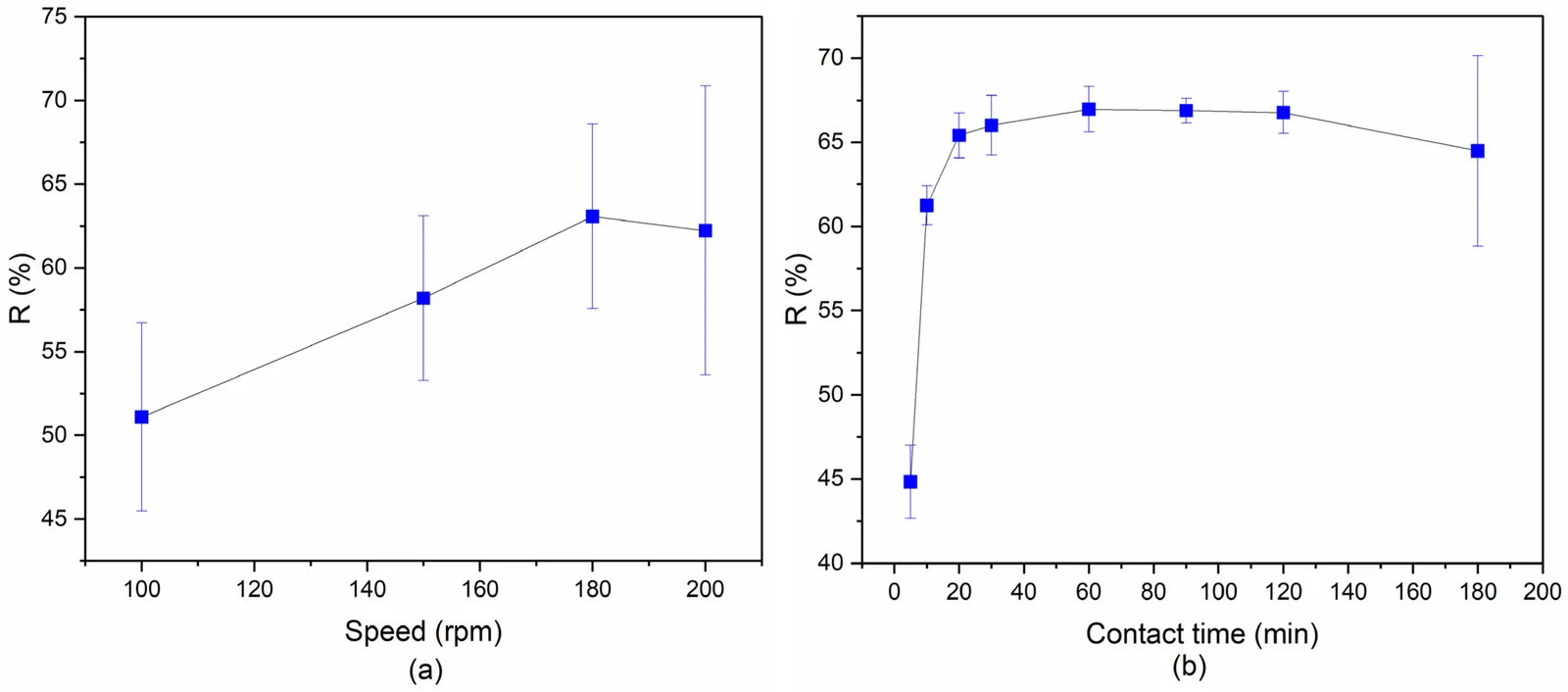
Effects of (**a**) agitation speed and (**b**) contact time on Cr(VI) removal by Fe_3_O_4_@eggshell NC under batch conditions. Blue squares represent mean values, and error bars indicate the standard deviation of three measurements (n = 3).

Agitation speed and contact time were examined together because both parameters govern the transport conditions under which Cr(VI) reaches the active sites of the adsorbent. Within the range of 100–200 rpm (Figure 9a), removal increased from 51.10% to 63.10% as the agitation speed rose to 180 rpm, followed by a slight decline at 200 rpm. Although the differences among the tested speeds were moderate, the results show that mixing conditions influenced adsorption performance and the stability of the solid suspension.

The increase in removal from 100 to 180 rpm indicates that stronger mixing improved particle dispersion and reduced the external mass-transfer resistance, thereby enhancing contact between dissolved Cr(VI) species and the Fe_3_O_4_@eggshell NC surface [60]. At 100 rpm, the lower removal was consistent with insufficient mixing, while at 150 rpm part of the material still settled at the bottom of the flask. By contrast, 180 rpm maintained the adsorbent in a homogeneous suspension without visible sedimentation. Further increasing the speed to 200 rpm did not improve removal and instead promoted partial adhesion of the material to the flask walls, which likely disturbed suspension uniformity. Similar behavior has been reported in other Cr(VI) adsorption systems. Redwan et al. [61] found that removal improved as mixing increased, only up to a threshold, beyond which performance declined, while Bejarano-Meza et al. [62] identified agitation speed as a key operational variable in magnetic nanoparticle systems and reported 140 rpm as the most favorable condition. Under the present conditions, 180 rpm provided the most suitable hydrodynamic regime.

The time-course results showed a rapid increase in removal during the initial stage of adsorption as presented in Figure 9b, from 44.84% at 5 min to 66.96% at 60 min. Thereafter, the response remained essentially unchanged at 90 min (66.88%) and 120 min (66.76%), followed by a slight decrease at 180 min (64.48%). This profile is characteristic of fast initial uptake while the most accessible surface sites remain available, followed by a progressive slowdown as these sites become occupied and the system approaches equilibrium. Comparable behavior has been reported for magnetite-containing adsorbents and related composites, which typically exhibit a rapid early stage followed by a plateau near equilibrium [44,57]. Since no meaningful improvement occurred beyond 60 min, this contact time was selected for the subsequent experiments. The slight decrease at 180 min, together with the greater associated variability, further indicates that prolonged contact did not provide a practical advantage under the selected conditions.

The results support the selection of 180 rpm and 60 min as the operating conditions that best balanced Cr(VI) removal, suspension stability, and process efficiency.

#### 2.4.3. Adsorbent Loading and Concentration-Dependent Performance

**Figure 10:**
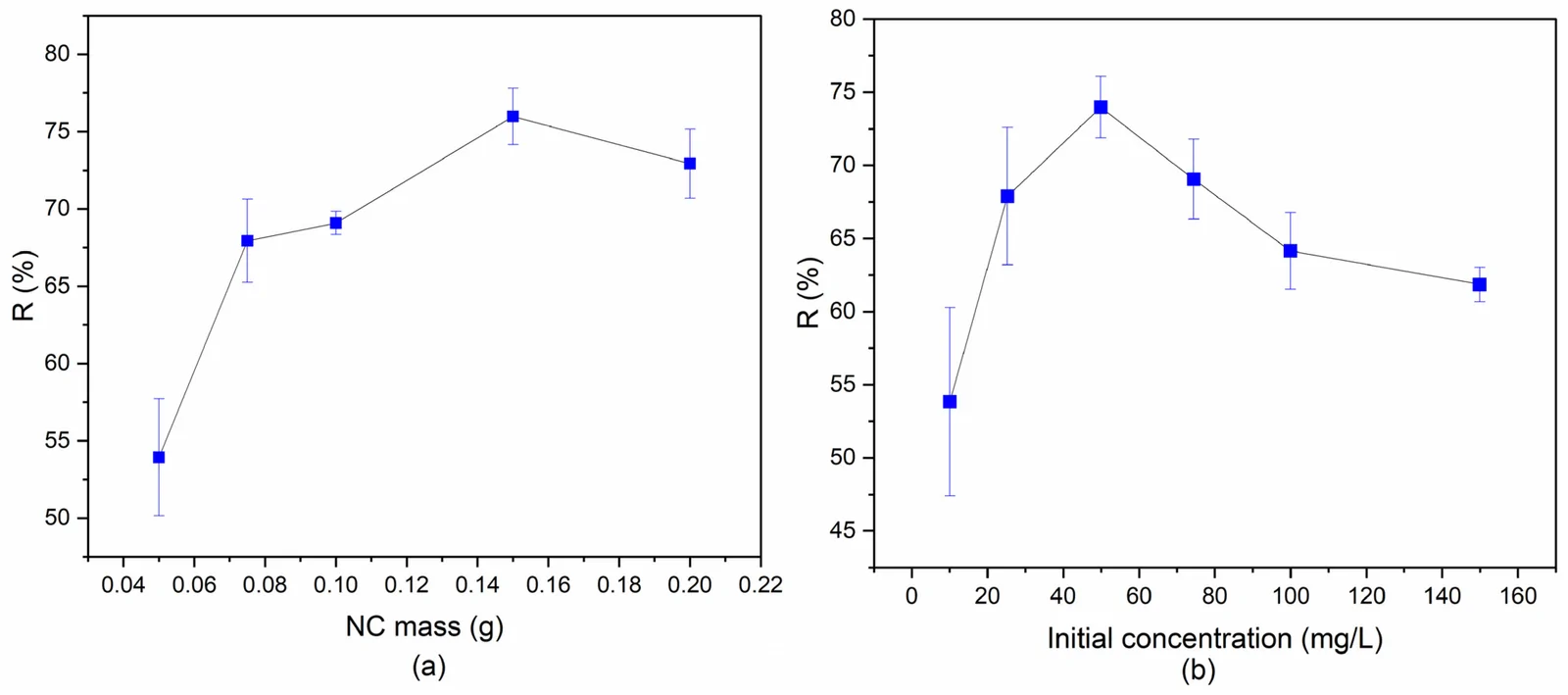
Effects of (**a**) adsorbent dosage and (**b**) initial Cr(VI) concentration on batch adsorption by Fe_3_O_4_@eggshell NC. Blue squares represent mean values, and error bars indicate the standard deviation of three measurements (n = 3).

The adsorption response was strongly influenced by adsorbent dosage and initial Cr(VI) concentration, as both parameters determine the balance between available surface sites and contaminant loading (Figure 10a,b). Increasing the adsorbent mass from 0.05 to 0.15 g improved removal efficiency from 53.93% to 75.98%, whereas a further increase to 0.20 g led to a slight decline to 72.93%. The improvement observed up to 0.15 g is attributable to the greater availability of active sites and the larger effective surface area accessible for interaction with Cr(VI) species. By contrast, the reduction at 0.20 g suggests that increasing the solid loading beyond the optimum did not enhance adsorption proportionally. This behavior is commonly associated with particle aggregation [63] or partial overlap of adsorption sites [23], both of which could reduce the fraction of surface effectively exposed to the solution.

A comparable balance emerged when the initial Cr(VI) concentration was varied from 10 to 150 mg/L. Removal efficiency increased from 53.83% at 10 mg/L to a maximum of 73.97% at 50 mg/L. Beyond this concentration, removal decreased to 69.05% at 75 mg/L, 64.16% at 100 mg/L, and 61.85% at 150 mg/L. The initial improvement indicates that a stronger concentration gradient enhanced mass transfer and increased the probability of interaction between Cr(VI) species and the active surface, as also noted by Segneanu et al. [44]. Once the initial concentration exceeded 50 mg/L, however, the fixed amount of adsorbent no longer provided sufficient sites to accommodate the higher contaminant load, and progressive saturation became more evident, leading to a lower fraction of Cr(VI) removed from solution despite the greater driving force for adsorption.

These results indicate that adsorption performance was controlled by the balance between site availability and solute loading. Under the selected experimental conditions, 0.15 g of adsorbent and an initial Cr(VI) concentration of 50 mg/L provided the most suitable operating combination for the subsequent equilibrium studies.

### 2.5. Adsorption Kinetics Evaluation

**Figure 11:**
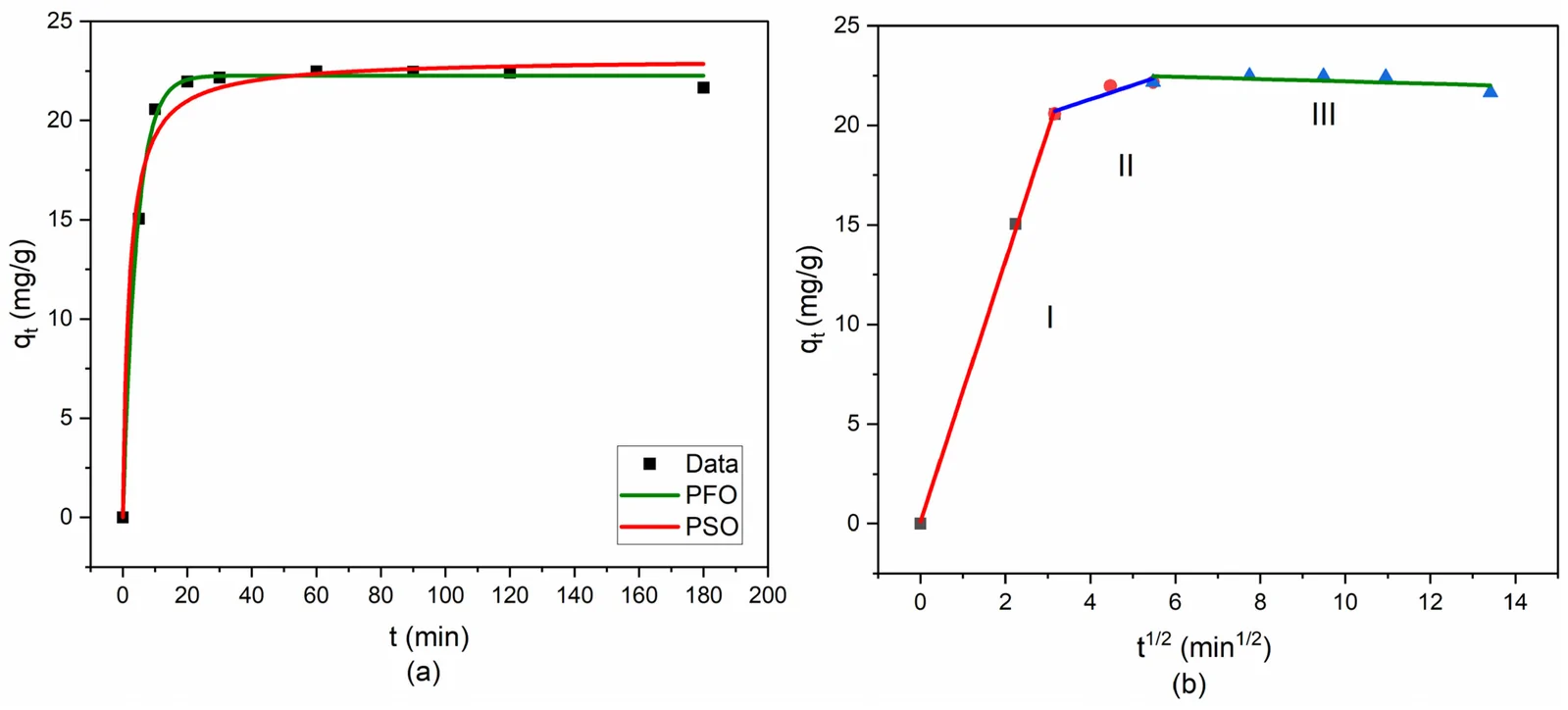
Kinetic modelling of Cr(VI) adsorption by Fe_3_O_4_@eggshell NC: (**a**) PFO and PSO fits; (**b**) intraparticle diffusion plot showing three adsorption regimes: I, external surface adsorption; II, intraparticle diffusion; and III, equilibrium stage.

The kinetic data were fitted with the pseudo-first-order (PFO), pseudo-second-order (PSO) and intraparticle diffusion models (Figure 11a,b) to identify the main rate-controlling features of Cr(VI) uptake by Fe_3_O_4_@eggshell NC. Among the evaluated models, the PFO provided the best description of the experimental data (Table 3), as indicated by its higher coefficient of determination (R^2^ = 0.9982) and lower reduced chi-square value (χ^2 red^ = 0.1117) compared with the PSO model (R^2^ = 0.9851; χ^2 red^ = 0.9395). In addition, the PFO-calculated equilibrium adsorption capacity (q_e_ = 22.2701 mg/g) was very close to the experimental value (q_e,exp_ = 22.4812 mg/g), further supporting the suitability of this model (Figure 11a).

The superior fit of the PFO model indicates that Cr(VI) uptake followed a first-order kinetic behavior, characterized by a rapid initial adsorption rate that progressively decreased as the system approached equilibrium. This kinetic result should not be interpreted as direct evidence of a specific adsorption mechanism, since PFO and PSO models are empirical descriptions of adsorption kinetics. In contrast, PSO kinetics have been reported for Cr(VI) adsorption on related heterogeneous composite adsorbents, including magnetized eggshell [57], an eggshell–termite mound composite [64], and Fe_3_O_4_-based nanoparticles supported on an organic matrix [65].

To further examine the mass-transfer pathway, the Weber–Morris intraparticle diffusion model was applied, as this approach can complement conventional kinetic analysis by identifying successive stages in the adsorption process [66]. The q_t_ versus t^1/2^ plot showed three distinct regions as seen in Figure 11b, indicating that Cr(VI) uptake did not occur through a single diffusion-controlled step. As summarized in Table 4, Region I exhibited the highest diffusion rate constant, with k_id_ of 6.5485 mg/(g min^1/2^), a near-zero intercept (C = 0.0924 mg/g), and an excellent fit (R^2^ = 0.9993). This stage is consistent with rapid uptake associated with external mass transfer and adsorption on the most accessible surface sites. Region II showed a much lower slope, with k_id_ of 0.7098 mg/(g min^1/2^), together with a larger intercept of 18.4678 mg/g and R^2^ of 0.8933, which points to a slower stage associated with diffusion toward less accessible domains of the adsorbent. By contrast, Region III displayed a very small negative slope (k_id_ = −0.0587 mg/(g min^1/2^)) and a poor fit (R^2^ = 0.2834), indicating that this segment does not represent a physically meaningful intraparticle diffusion stage and is better interpreted as the approach to equilibrium.

The fact that the linear segments did not pass through the origin indicates that intraparticle diffusion was not the only rate-limiting step and that film diffusion, transport within the adsorbent structure, and surface interactions may also have contributed to the overall adsorption rate [25].

The present results support a multi-step uptake pathway in which Cr(VI) removal was initially dominated by rapid surface adsorption and external mass transfer, followed by a slower transport stage toward less accessible domains of the adsorbent. At longer contact times, the system approached equilibrium and diffusion no longer appeared to govern the overall rate.

This kinetic behavior is consistent with the hybrid architecture of the nanocomposite. The Fe_3_O_4_ phase likely contributes to the surface sites associated with the rapid initial uptake, whereas the CaCO_3_-rich eggshell-derived matrix may provide a porous framework that supports transport toward less accessible domains during the subsequent stage of adsorption.

### 2.6. Equilibrium Isotherm Analysis

**Figure 12:**
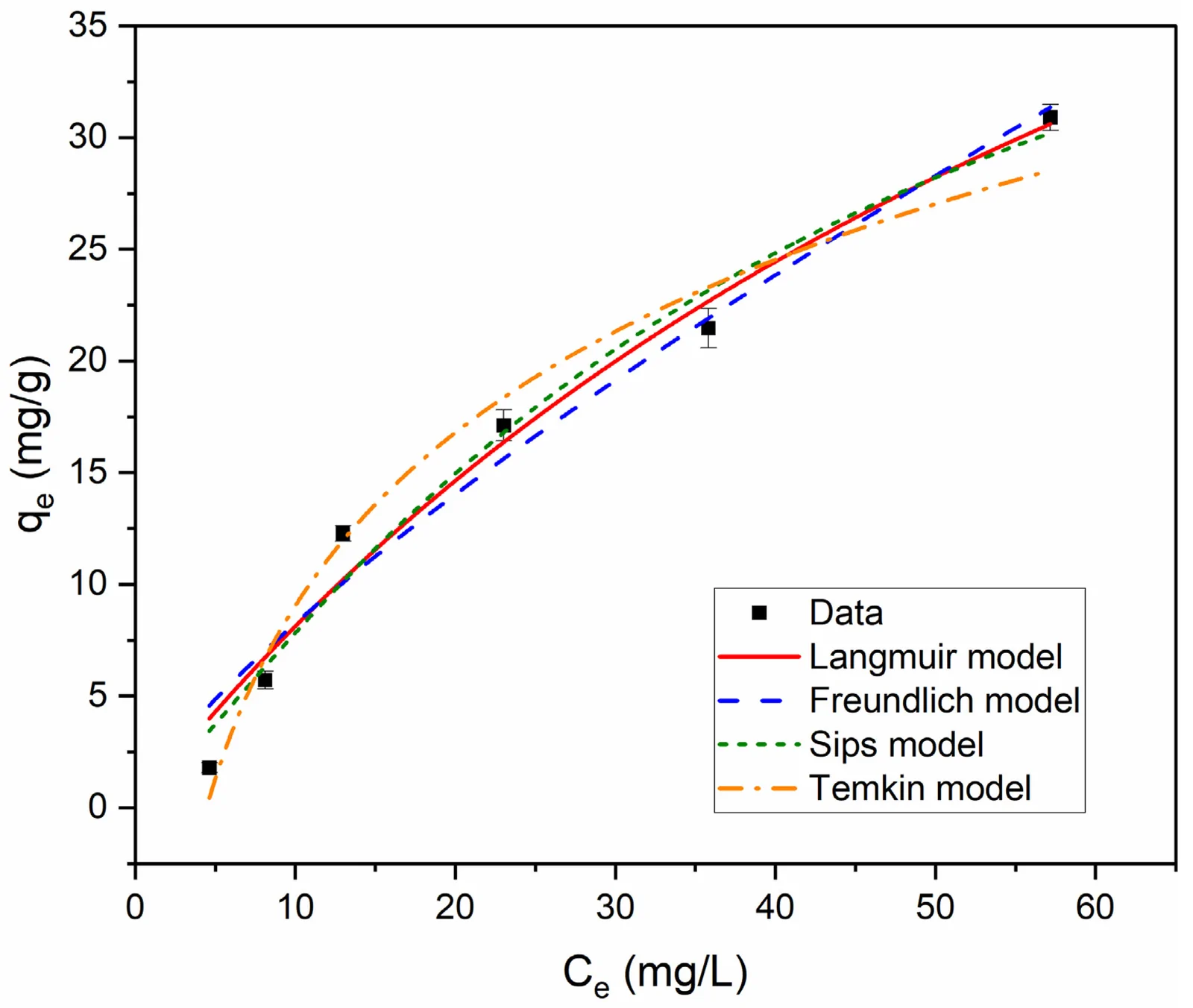
Non-linear isotherm fits for Cr(VI) adsorption on Fe_3_O_4_@eggshell NC. Error bars indicate the standard deviation of three measurements (n = 3).

The experimental equilibrium data were analyzed by non-linear regression using two-parameter (Langmuir, Freundlich, and Temkin) and three-parameter (Sips) isotherm models to describe the adsorption behavior of Cr(VI) on Fe_3_O_4_@eggshell NC. The fitted curves are presented in Figure 12, and the corresponding parameters are listed in Table 5. Among the evaluated models, the Sips equation showed the highest R^2^ (0.9803; χ^2^ = 3.7286), whereas Langmuir yielded the lowest χ^2^ value (3.0714; R^2^ = 0.9783), indicating comparable overall performance of these models. Temkin also showed a good fit (R^2^ = 0.9753; χ^2^ = 3.3540), whereas Freundlich showed the lowest, though still acceptable, fit (R^2^ = 0.9701; χ^2^ = 4.2444).

The Sips model, which provided the highest R^2^ value, together with the estimated capacity (q_s_ = 50.8142 mg/g), suggests that the adsorption process involved a non-uniform distribution of sites while progressively approaching saturation. This interpretation is consistent with the heterogeneous surface expected for the nanocomposite. In addition, the Sips heterogeneity exponent (n_s_ = 1.1960) indicates a moderate deviation from ideal Langmuir behavior, suggesting that surface heterogeneity was present [67], although not strongly pronounced over the investigated concentration range.

Although the Langmuir model also described the data well, its estimated maximum adsorption capacity, q_max_ = 74.0599 mg/g, should be interpreted with caution. The equilibrium data did not provide clear experimental evidence of ideal monolayer saturation within the investigated concentration range, indicating that the Langmuir capacity was at least partially influenced by extrapolation. Thus, the good Langmuir fit does not necessarily imply an ideal monolayer adsorption mechanism, but rather that the equilibrium data approached a saturation-type behavior over the explored range. A Langmuir-dominated behavior was reported for a Fe_3_O_4_-eggshell biocomposite [23]. In the present system, however, the Sips model provides a more informative interpretation, since it accounts for surface heterogeneity at low concentrations while retaining a Langmuir-type saturation limit at higher concentrations.

The Freundlich model also provided a reasonable description of the data, which supports the presence of surface heterogeneity. In particular, the value of 1/n (0.7660) indicates favorable adsorption and a non-uniform distribution of adsorption energies across the adsorbent surface. This is physically plausible for Fe_3_O_4_@eggshell NC, given the coexistence of Fe-based reactive sites, carbonate domains, and surface functional groups derived from the green synthesis route. Freundlich-type behavior has been reported for other complex Cr(VI) adsorbents [24,44].

The Temkin model showed a comparably good fit, consistent with the possible contribution of interaction effects to the equilibrium adsorption response. In particular, the A_T_ (0.2247 L/mg) represents the affinity term in the Temkin equation, whereas B_T_ (11.7505 mg/g) governs the variation in adsorption capacity with the logarithm of equilibrium concentration.

Taken together, these results indicate that Cr(VI) adsorption on Fe_3_O_4_@eggshell NC is better described by a heterogeneous equilibrium model with saturation tendencies than by a strictly ideal monolayer mechanism. Under these conditions, the Sips model provides a useful interpretation of the equilibrium behavior.

### 2.7. Thermodynamic Evaluation

The thermodynamic behavior of Cr(VI) adsorption on Fe_3_O_4_@eggshell NC was evaluated at a fixed initial concentration of 50 mg/L, selected as the most suitable operating concentration based on the preceding concentration-effect study. The analysis was conducted using equilibrium data collected at 293, 298, 308, 318, and 328 K. The thermodynamic constant (K_c_) was calculated at each temperature and used to determine the standard thermodynamic parameters ΔG°, ΔH°, and ΔS°. As shown in Figure S5, the Van’t Hoff plot exhibited a linear relationship between lnK_c_ and 1/T.

The resulting thermodynamic parameters are listed in Table 6. Since K_c_, as defined in Equation (9), depends on the initial concentration, the thermodynamic parameters reported here correspond specifically to the investigated condition of C_0_ = 50 mg/L and should not be extrapolated to the other initial concentrations evaluated in the equilibrium isotherm study.

Under this experimental condition, the adsorption process showed a clear temperature dependence. The values of ΔG° were negative at 293, 298, and 308 K, indicating that Cr(VI) uptake was thermodynamically favored within this interval. At 318 and 328 K, however, ΔG° became slightly positive, reflecting a reduced tendency for adsorption as temperature increased.

This response is consistent with the negative enthalpy change obtained from the Van’t Hoff analysis. The ΔH° value of −16.792 kJ/mol indicates an exothermic adsorption process under the investigated conditions. At C_0_ = 50 mg/L, increasing temperature progressively reduced the thermodynamic driving force for adsorption, which explains the loss of spontaneity above 308 K. This behavior differs from that reported by Segneanu et al. [44], who observed an endothermic response in a Fe_3_O_4_-containing eggshell/fly ash composite, highlighting the strong influence of adsorbent composition and structure on thermodynamic behavior.

The entropy change provides additional insight into the interfacial rearrangements accompanying adsorption. The negative ΔS° value (−52.813 J/mol K) indicates a decrease in randomness at the solid–liquid interface as Cr(VI) species were transferred from the aqueous phase to the adsorbent surface. This result is consistent with increased interfacial ordering and reduced mobility of the adsorbed species during uptake. A similar negative entropy change was reported by Ravi and Sundararaman [57] for Cr(VI) adsorption on Fe_3_O_4_-coated thermally treated eggshell.

At the investigated initial concentration of 50 mg/L, the thermodynamic parameters indicate that Cr(VI) adsorption was favorable up to 308 K and exothermic in nature, with the thermodynamic driving force progressively decreasing as temperature increased.

### 2.8. Proposed Mechanism of Cr(VI) Removal by Fe_3_O_4_@eggshell NC

**Figure 13:**
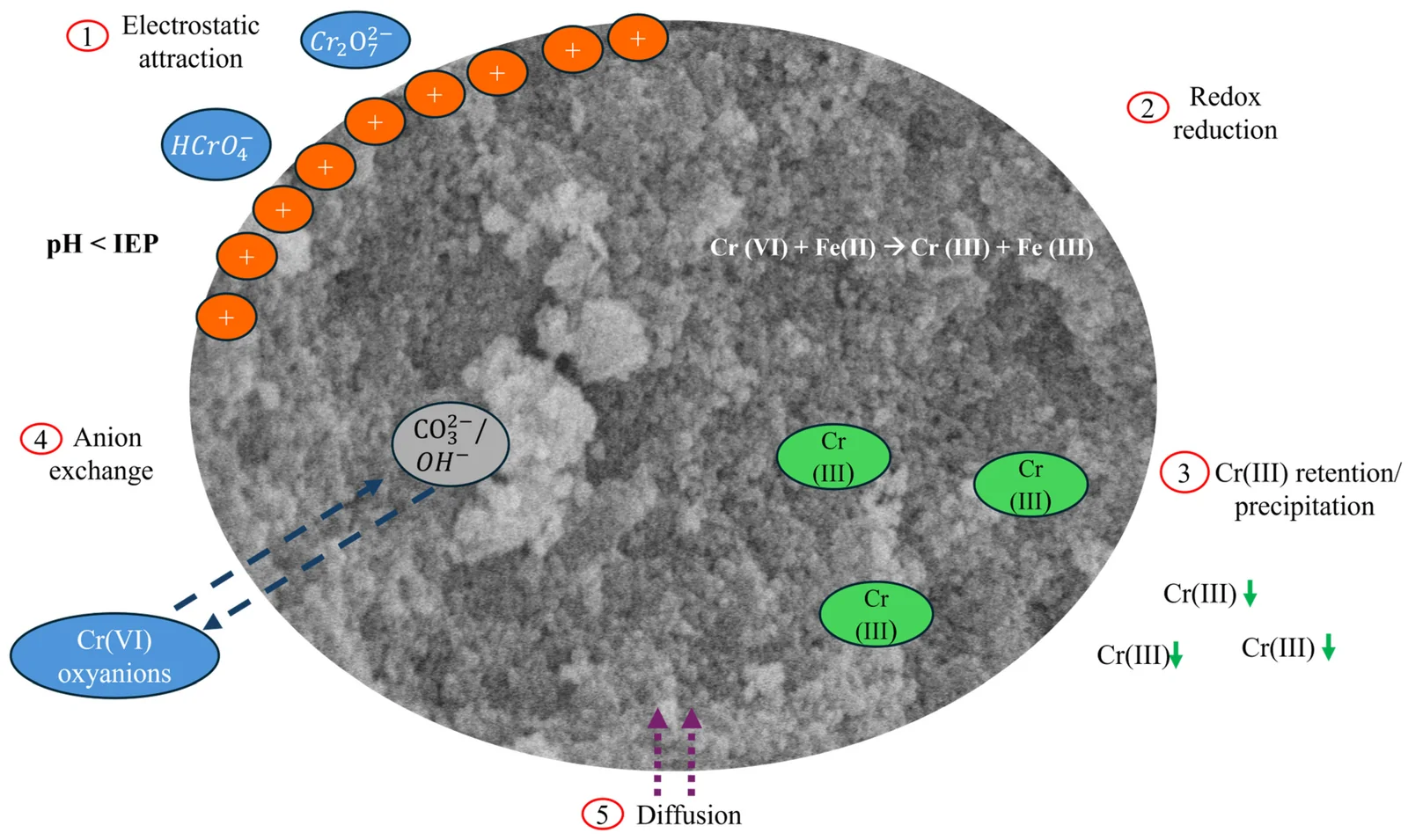
Proposed mechanism of Cr(VI) removal by Fe_3_O_4_@eggshell NC, including electrostatic attraction, redox reduction, Cr(III) retention/precipitation, anion exchange, and diffusion through the porous matrix. The “+” symbols denote positively charged surface sites, while the arrows indicate the direction of the proposed transport and reaction pathways.

Cr(VI) removal by Fe_3_O_4_@eggshell NC is best interpreted as a synergistic process involving multiple pathways rather than a single adsorption mechanism. The most plausible contributions include electrostatic attraction of anionic Cr(VI) species, redox reduction to Cr(III), subsequent retention or precipitation of Cr(III), anion exchange with carbonate- and hydroxyl-bearing surface groups, and diffusion within the porous matrix. The relative contribution of these processes depends on solution pH, chromium speciation, and the hybrid nature of the adsorbent, as schematically illustrated in Figure 13.

Under acidic conditions (pH 4), the initial uptake of Cr(VI) is likely driven by electrostatic attraction between dissolved chromium oxyanions (${HCrO}_{4}^{-}$ and ${Cr}_{2}O_{7}^{2-}$) and positively charged surface sites on the nanocomposite surface. The zeta potential supports this interpretation, since the nanocomposite exhibited a positive surface charge at pH 4 and an isoelectric point of 5.57, indicating that the surface remained protonated below this value and was therefore favorable for anion uptake. Such behavior may be associated with protonated sites from the Fe_3_O_4_ phase, residual organic functionalities introduced during green synthesis, and the eggshell-derived matrix. Yadav et al. [16] similarly reported that surface functional groups in plant-extract-functionalized magnetic systems can provide additional interaction sites and enhance the initial attraction of Cr(VI). A further contribution may arise from the carbonate-rich fraction of the material, since calcium-containing surface domains have been associated with Cr(VI) binding in magnetized eggshell systems [57].

In addition, the Fe_3_O_4_ phase may actively participate in chromium transformation through a redox pathway. Because magnetite contains both Fe(II) and Fe(III), Fe(II)-bearing sites can serve as electron donors and promote reduction of Cr(VI) to Cr(III), particularly in acidic media [68]. This transformation is environmentally significant because Cr(III) is less toxic and less mobile than Cr(VI).

Once formed, Cr(III) may be immobilized through surface retention and/or precipitation. Specific interactions with hydroxyl-, carboxyl-, or other oxygen-containing groups available on the eggshell-derived matrix and on the outer domains of the hybrid material may promote retention of Cr(III) on the adsorbent surface [4,24]. Under favorable local conditions, Cr(III) may precipitate as poorly soluble hydroxide species, further contributing to chromium immobilization. These pathways are not mutually exclusive and are consistent with the heterogeneous surface chemistry expected for Fe_3_O_4_@eggshell NC.

Furthermore, Ajab et al. [23] described magnetized eggshell materials as porous matrices with ion-exchange capacity, in which chromium species can interact with charged surface sites. The presence of carbonate and hydroxyl groups has been associated with Cr(VI) adsorption in eggshell-based materials [69]. Accordingly, anion exchange between Cr(VI) oxyanions and surface carbonate/hydroxyl groups may represent a complementary mechanism in the present material.

Finally, the porous architecture of the eggshell-derived matrix may facilitate transport of chromium species toward internal regions of the adsorbent, where additional interactions can occur [37,69]. This interpretation agrees with the multi-step kinetic behavior, in which rapid initial uptake was followed by a slower transport stage toward less accessible domains.

These considerations support a multisite and multiphase mechanism for Cr(VI) removal by Fe_3_O_4_@eggshell NC. The Fe_3_O_4_ phase may contribute redox-active and adsorption-related surface sites while also enabling magnetic recovery, whereas the CaCO_3_-rich eggshell matrix provides structural support, porosity, exchangeable surface groups, and additional interaction domains. The overall behavior is therefore consistent with that reported for other heterogeneous Fe_3_O_4_-based biogenic composites, in which electrostatic attraction, redox conversion, specific surface retention, ion exchange, diffusion, and precipitation act in a complementary manner to immobilize chromium from aqueous media [4,68,70].

### 2.9. Comparison with Selected Magnetic Adsorbents Reported for Cr(VI) Removal

Table 7 compares selected magnetic adsorbents reported for Cr(VI) removal under different experimental conditions. Because adsorption performance depends on variables such as pH, initial Cr(VI) concentration, adsorbent dosage, contact time, and material composition, the reported values should be interpreted in the context of each study.

The literature shows a broad range of Cr(VI) removal efficiencies and adsorption capacities for magnetite composite-based materials. Within this context, the Fe_3_O_4_@eggshell NC developed in the present study can be situated among magnetic adsorbents prepared from residual matrices and evaluated under acidic conditions. Under the selected operating conditions (pH 4, 0.15 g in 50 mL, 50 mg/L, 60 min), the material achieved 75.98% removal and a model-estimated adsorption capacity of 50.81 mg/g over the investigated equilibrium concentration range. Notably, this performance was achieved at pH 4, whereas some related systems have been evaluated under more strongly acidic conditions. In addition, the nanocomposite was obtained through a green route based on *Passiflora ligularis* extract and uncalcined eggshell waste.

### 2.10. Regeneration Studies

The reusability of Fe_3_O_4_@eggshell NC was evaluated over five consecutive adsorption–regeneration cycles using 0.1 M NaOH and 0.5 M NaCl as regenerating agents (Figure S6). In both cases, adsorption performance generally declined with cycle number, indicating incomplete regeneration and progressive loss of accessible active sites. Such deterioration is commonly associated with residual chromium retention, irreversible site occupation, partial pore blockage, and surface alterations induced by repeated regeneration [71,72].

A clear difference was observed between the two regenerants. The material regenerated with 0.1 M NaOH maintained higher adsorption percentages over the reuse sequence, with only minor variation during the first three cycles and a more marked decline thereafter. In contrast, the NaCl-regenerated material showed a steady loss of performance from the second cycle onward and reached its lowest adsorption values in the final cycles. This result suggests that alkaline treatment restored the adsorption performance more effectively, which is consistent with the ability of hydroxide ions to promote desorption of previously retained anionic species under basic conditions [73]. For Cr(VI) removal, NaOH has been identified as one of the most effective regenerating solutions for iron oxide-based adsorbents [74]. By comparison, NaCl likely acted mainly through ionic-strength and chloride-competition effects, which were less effective in recovering the active adsorption surface [75].

The same trend was observed when adsorption performance was normalized to the first cycle (Table S6). For the material regenerated with 0.1 M NaOH, capacity retention remained high during the first three cycles (97.15–95.94%) and then decreased to 65.20% and 67.60% in cycles 4 and 5, respectively. In contrast, the sample regenerated with 0.5 M NaCl showed a continuous decline from 84.67% in cycle 2 to 51.66% in cycle 5. These results indicate that Fe_3_O_4_@eggshell NC exhibited moderate reusability over the first three cycles, with better performance under alkaline regeneration.

## 3. Materials and Methods

All reagents were of analytical grade and purchased from Merck KGaA (Darmstadt, Germany), including iron(II) chloride tetrahydrate (FeCl_2_·4H_2_O), iron(III) chloride hexahydrate (FeCl_3_·6H_2_O), sodium hydroxide (NaOH), hydrochloric acid (HCl), potassium dichromate (K_2_Cr_2_O_7_), 2,2-diphenyl-1-picrylhydrazyl (DPPH), ethanol (96%, *v*/*v*), 1,5-diphenylcarbazide (DPC), sulfuric acid (H_2_SO_4_), sodium chloride (NaCl) and acetone. Ultrapure water was obtained from a Milli-Q Integral water purification system (Millipore SAS, Molsheim, France), whereas deionized water was obtained from a BIOBASE water purification system (Jinan, China).

*Passiflora ligularis* peels were collected from local markets in Arequipa, Peru, where vendors set aside fruits with visual defects or discoloration that prevented their commercialization. The peels were manually cleaned to remove the inner mesocarp, washed with tap water followed by deionized water, air-dried at room temperature for 24 h, and dried at 40 °C for 72 h. The dried material was ground and sieved through a No. 100 mesh. This particle size was adequate for extract preparation and subsequent filtration. The material was stored in sealed containers at 4 °C, protected from light until use. Chicken eggshells were obtained from a local bakery. The processing method was adapted from Besharati et al. [25]. The material was washed, air-dried for 12 h, and oven-dried at 100 °C for 2 h. It was then ground, sieved (No. 200 mesh) and additionally dried at 100 °C for 24 h to remove residual moisture. The obtained eggshell powder was stored in airtight containers to prevent moisture absorption and contamination.

### 3.1. Passiflora ligularis Extract Preparation

A 2^3^ factorial design was employed to evaluate the effect of solvent concentration (A), extraction temperature (B), and extraction time (C), as seen in Table S1. The antioxidant capacity, measured by the DPPH assay, was selected as the response variable. Each experimental run was performed in duplicate, and a central point (CP) was carried out in triplicate to assess possible curvature in the response surface. Factorial effects, interactions, and design curvature were evaluated by ANOVA, with statistical significance assessed at *p* < 0.05.

Ultrasound-assisted extraction was carried out using a UC-20A ultrasonic cleaner (BIOBASE, Jinan, China) with a 1:10 ratio of *Passiflora ligularis* peel powder to solvent. After extraction, the mixture was allowed to stand overnight, filtered through Whatman No. 42 filter paper (Cytiva, Marlborough, MA, USA), and concentrated by gentle heating until one-half of the initial volume was reached. The extract was then adjusted to a final volume of 100 mL using the same ethanol concentration employed in the extraction step. Samples were stored at 4 °C in amber bottles and used within 15 days of preparation.

Antioxidant capacity was determined using the DPPH radical-scavenging assay. A 0.1 mM DPPH solution was prepared in 96% ethanol and protected from light during handling. For the assay, 100 μL of each extract was mixed with 3.9 mL of the DPPH solution in centrifuge tubes and incubated in the dark at room temperature for 30 min. Absorbance was then measured at 517 nm using a GENESYS UV–Vis spectrophotometer (Thermo Scientific, Madison, WI, USA). A DPPH solution without extract was used as the control. The percentage of DPPH radical inhibition was calculated with Equation (2):

(2) $$\%\;inhibition=\frac{A_{control}-A_{sample}}{A_{control}}\times 100$$

### 3.2. Fe_3_O_4_@eggshell NC Synthesis Procedure

The Fe_3_O_4_@eggshell NC was synthesized by a two-step green coprecipitation method adapted from Omidi et al. [41]. The experimental strategy followed a 2^2^ full factorial design, in which synthesis pH and eggshell mass were selected as independent variables (Table S2). Three central points were included to estimate experimental error and assess response curvature. The specific surface area (m^2^/g), determined by BET analysis using a Gemini VII 2390t analyzer (Micromeritics Instrument Corporation, Norcross, GA, USA), was selected as the response variable. The effects of pH, eggshell mass, their interaction, and design curvature were evaluated by ANOVA, with statistical significance established at *p* < 0.05.

For each run, 0.8586 g of FeCl_2_·4H_2_O and 2.3347 g of FeCl_3_·6H_2_O were separately dissolved in 10 and 20 mL of ultrapure water, respectively, and then combined to provide an Fe^2+^:Fe^3+^ molar ratio of 1:2. The solution was stirred at 300 rpm on an AREX hotplate magnetic stirrer (VELP Scientifica Srl, Usmate, Italy) under continuous nitrogen flow for 15 min. The reaction temperature was maintained at room temperature (approximately 21 °C). Subsequently, 30 mL of *Passiflora ligularis* peel extract was added dropwise, and stirring was continued for a further 15 min. The iron precursor solution and the extract were used at a 1:1 volume ratio.

The suspension pH was initially adjusted to 6.5 through the controlled addition of 2 M NaOH. At this stage, the predetermined amount of eggshell powder was introduced in accordance with the experimental design, corresponding to nominal magnetite-to-eggshell mass ratios of 1:1, 1:1.5 and 1:2.

The pH was then further adjusted to the value specified in the factorial design to complete the formation of magnetite nanoparticles. The reaction mixture was maintained under constant stirring at 600 rpm for 1 h. The formation of the Fe_3_O_4_@eggshell NC was evidenced by the development of an intense black coloration.

**Figure 14:**
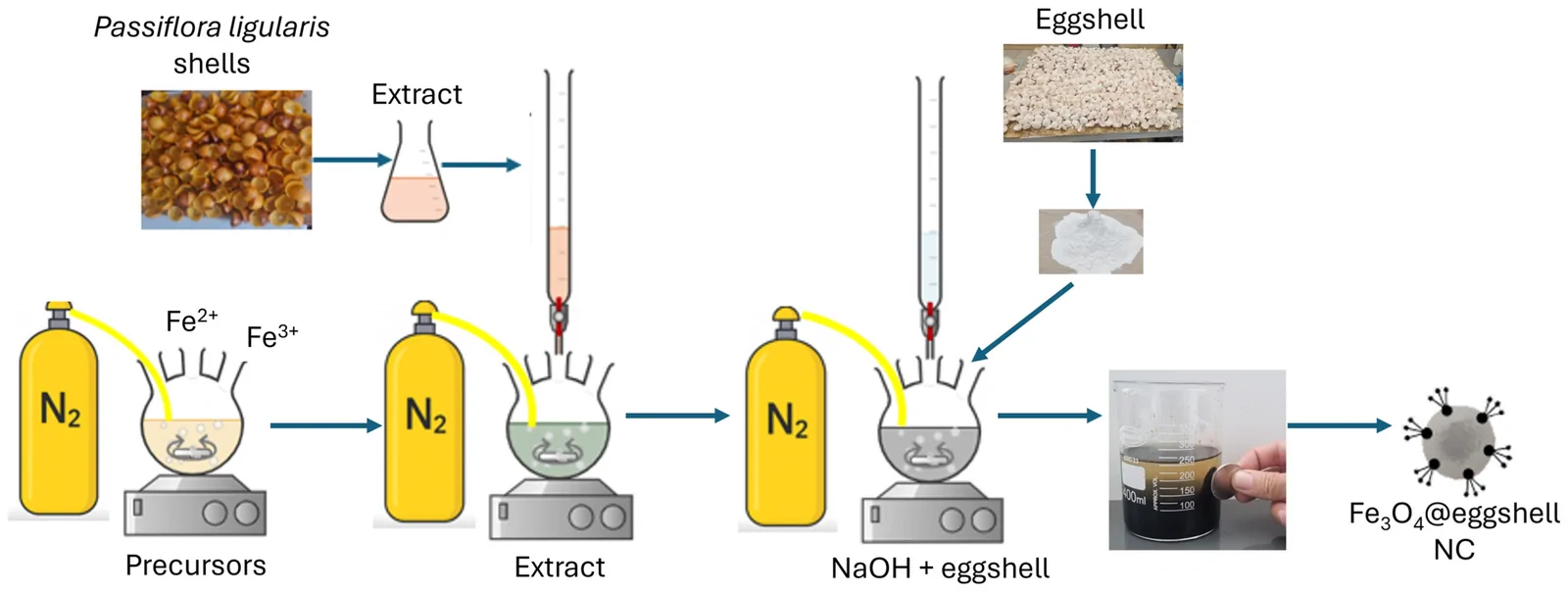
Schematic representation of the biogenic synthesis of Fe_3_O_4_@eggshell NC. Arrows indicate the sequence of synthesis steps and material additions.

After the synthesis, the suspension was sonicated for 15 min in a Bransonic 2800 ultrasonic bath (Branson Ultrasonics Corporation, Danbury, CT, USA). The supernatant was removed by decantation. The solid was then washed 6 times by centrifugation at 4000 rpm for 10 min in a Z 306 centrifuge (HERMLE Labortechnik GmbH, Wehingen, Germany), first with ultrapure water and then with ethanol (96%, *v*/*v*). The resulting NC was dried in an oven (Nabertherm GmbH, Lilienthal, Germany) at 80 °C for 15 h, ground and stored in microcentrifuge tubes inside a desiccator. Figure 14 shows the synthesis procedure of Fe_3_O_4_@eggshell NC and outlines the main stages of the process.

### 3.3. Characterization of Fe_3_O_4_@eggshell NC

The physicochemical properties of the synthesized Fe_3_O_4_@eggshell NC were characterized using complementary analytical techniques. FTIR spectra were recorded using an INVENIO spectrometer (Bruker Optics GmbH & Co. KG, Ettlingen, Germany) equipped with a Platinum ATR accessory over the 4000–400 cm^−1^ range to identify surface functional groups. Crystalline phases were determined by XRD with a MiniFlex 600 diffractometer (Rigaku Corporation, Akishima, Tokyo, Japan) equipped with Cu Kα radiation (λ = 0.15406 nm). Diffractograms were recorded in the 2θ range of 15–85°, with a step size of 0.02° and a scan rate of 2°/min. The crystallite size of the magnetite phase was estimated using the Scherrer equation, with a shape factor (K) of 0.89, assuming approximately spherical crystallites. Quantitative phase analysis was performed by Rietveld refinement using the reference structures of calcite (COD 96-900-9668) and magnetite (COD 96-900-5838).

Eggshell morphology and elemental composition were analyzed by FE-SEM using a Scios 2 FIB-SEM (Thermo Fisher Scientific, Brno, Czech Republic) coupled with an energy-dispersive X-ray spectroscopy (EDS) detector (EDAX Inc., Mahwah, NJ, USA). Fe_3_O_4_ NPs and the Fe_3_O_4_@eggshell NC were analyzed by SEM using an S-4800 field-emission scanning electron microscope (Hitachi High-Technologies, Tokyo, Japan) equipped with an UltraDry EDX detector (Thermo Fisher Scientific, Madison, WI, USA). TEM analysis was performed on an EM 109T transmission electron microscope (Carl Zeiss, Jena, Germany) equipped with an ES1000W digital camera (Gatan Inc., Pleasanton, CA, USA) to evaluate nanoparticle morphology and size distribution. Samples were dispersed in ethanol, deposited onto carbon-coated copper grids, and dried at room temperature. Particle diameters were measured for a total of 200 nanoparticles using DigitalMicrograph software version 3.7.1 (Gatan Inc., Pleasanton, CA, USA), and the resulting size distribution was fitted to a log-normal function. 

Specific surface area and pore characteristics were determined from nitrogen adsorption–desorption isotherms using a Gemini VII 2390t analyzer. Before analysis, the samples were degassed under helium (99.999%) at 100 °C for 3 h. The specific surface area was calculated using the BET method, while pore size distribution was derived from the desorption branch using the Barrett–Joyner–Halenda (BJH) method. Zeta potential measurements were performed with a Zetasizer Nano ZS (Malvern Instruments Ltd., Malvern, UK) to evaluate surface charge and colloidal stability in aqueous suspension. Thermal stability was assessed by TGA using a LABSYS evo system (SETARAM, Caluire, France) under a nitrogen atmosphere from 25 to 1000 °C and a heating rate of 10 °C/min.

Magnetic properties, including saturation magnetization (M_S_), remanent magnetization (M_R_), and coercivity (H_C_), were evaluated using a Lake Shore LS80 system (Lake Shore Cryotronics, Inc., Westerville, OH, USA) under an applied magnetic field of up to 3 T at room temperature. Mössbauer spectra were recorded in transmission geometry using a ^57^Co source embedded in a Rh matrix. Low-temperature spectra (down to 15 K) were obtained using a Janis closed-cycle cryostat (Lake Shore Cryotronics, Inc., Westerville, OH, USA). Temperature-dependent magnetization was also recorded under ZFC and FC protocols between 50 and 400 K under an applied field of 10 Oe.

### 3.4. Cr(VI) Determination by DPC Colorimetry

Cr(VI) concentrations were determined by UV–Vis spectrophotometry using a Genesys 150 spectrophotometer and the DPC colorimetric method under acidic conditions, following procedures commonly applied in related Cr(VI) adsorption studies [76]. A 1000 mg/L Cr(VI) stock solution was prepared from K_2_Cr_2_O_7_ and working standards in the range of 0.2–1.4 mg/L were obtained by serial dilution with deionized water. Before analysis, samples were adjusted to a pH of approximately 2 with 0.2 N H_2_SO_4_, followed by the addition of 200 μL of DPC reagent (5 mg/mL in acetone). After 8 min of color development, the absorbance of the red-violet chromium–DPC complex was measured at 540 nm against a reagent blank using 1 cm quartz cuvettes. Residual Cr(VI) was quantified by external calibration, and samples were diluted when necessary to fall within the working range.

The analytical performance of the method was evaluated from the calibration curve and replicate blank measurements. Linear calibration yielded the regression equation y = 0.9584× + 0.0063 with R^2^ = 0.9988. The limit of detection (LOD) and limit of quantification (LOQ) were estimated as 3.3σ/m and 10σ/m, respectively, where σ is the standard deviation of replicate reagent-blank signals and m is the slope of the calibration curve. Based on this approach, the LOD and LOQ were 0.0067 and 0.0204 mg/L, respectively.

### 3.5. Batch Adsorption Experiments

Batch adsorption tests were conducted to evaluate Cr(VI) removal by the Fe_3_O_4_@eggshell NC. All experiments were performed in triplicate using 50 mL of Cr(VI) solution at room temperature (approximately 20 °C). The initial pH was adjusted prior to adsorbent addition using 0.1 M HCl or 0.1 M NaOH. The effects of the main operating variables were assessed by varying the initial pH (3–7), agitation speed (100, 150, 180, and 200 rpm), contact time (0, 5, 10, 20, 30, 60, 90, 120, and 180 min), adsorbent mass (50, 75, 100, 150, and 200 mg), and initial Cr(VI) concentration (10, 25, 50, 75, 100, and 150 mg/L). The suspensions were agitated in a BJPX-ST68B thermostatic shaking incubator (BIOBASE, Jinan, China). At the end of each experiment, the adsorbent was magnetically separated using a cylindrical neodymium magnet for 2 min, and the supernatant was filtered through a 0.22 μm PVDF syringe filter. Residual Cr(VI) was then determined using the DPC UV–Vis method described in Section 3.4.

Removal efficiency (R) was calculated using Equation (3), where C_0_ and Ct are the initial and residual Cr(VI) concentrations (mg/L), respectively.

(3) $$R=\frac{C_{0}-C_{t}}{C_{0}}\times 100$$

The equilibrium adsorption capacity was calculated using Equation (4), where q_e_ denotes the amount of Cr(VI) adsorbed per unit mass of adsorbent at equilibrium (mg/g); C_e_ is the equilibrium Cr(VI) concentration in solution (mg/L), V is the solution volume (L), and m is the dry mass of adsorbent (g).

(4) $$q_{e}=\frac{\left(C_{0}-C_{e}\right)\times V}{m}$$

### 3.6. Adsorption Kinetics

Kinetic experiments were conducted to determine the time required to reach equilibrium and to describe the Cr(VI) uptake by the Fe_3_O_4_@eggshell NC at pH 4 and 180 rpm, using 75 mg of adsorbent at an initial Cr(VI) concentration of 50 mg/L. Kinetic data (qt vs. t) were fitted by non-linear regression to the PFO and PSO models (Equations (5) and (6)):

(5) $$q_{t}=q_{e}(1-e^{-k_{1}t})$$

(6) $$q_{t}=\frac{k_{2}q_{e}^{2}t}{{1+k}_{2}q_{e}t}$$

where q_t_ and q_e_ are the adsorption capacities at time t and at equilibrium (mg/g), k_1_ is the PFO rate constant (min^−1^), and k_2_ is the PSO rate constant (g/(mg min)).

To further examine mass-transfer limitations, the intraparticle diffusion model (Equation (7)) was also applied:

(7) $$q_{t}=k_{id}t^{1/2}+C$$

where k_id_ is the intraparticle diffusion rate constant (mg/(g min^1/2^)) and C is the intercept related to boundary-layer effects (mg/g). This model was interpreted by considering the different linear regions of the q_t_ vs. t ^1/2^ plot.

Model adequacy was evaluated using goodness-of-fit metrics (R^2^ and reduced χ^2^), and the best-fitting model was used to describe Cr(VI) uptake onto the Fe_3_O_4_@eggshell NC.

### 3.7. Equilibrium Isotherms

Equilibrium isotherm experiments were performed at pH 4 and 180 rpm using 150 mg of adsorbent with initial concentrations ranging from 10 to 150 mg/L. Isotherm analysis was performed to describe the relationship between the adsorption capacity at equilibrium (q_e_) and the equilibrium Cr(VI) concentration in solution (C_e_). The q_e_ vs. C_e_ dataset was fitted by non-linear regression to the Langmuir, Freundlich, Sips, and Temkin isotherm models, as summarized in Table 8, and fit quality was evaluated using R^2^ and χ^2^.

### 3.8. Thermodynamic Study

Thermodynamic experiments were carried out at 293, 298, 308, 318, and 328 K at pH 4 and 180 rpm, using 150 mg of Fe_3_O_4_@eggshell NC, an initial Cr(VI) concentration of 50 mg/L, and a contact time of 60 min. All experiments were performed in triplicate, and the reported results correspond to the mean values. The equilibrium adsorption capacity (q_e_) was calculated as described in Equation (4). The standard thermodynamic parameters, namely the Gibbs free energy change (∆G°), enthalpy change (∆H°), and entropy change (∆S°), were determined.

Since the equilibrium term used in thermodynamic calculations should be independent of the concentration units adopted [77,78], the dimensionless equilibrium constant (K_c_) for the present batch system was expressed according to Equation (8):

(8) $$K_{C}=\frac{K_{d}m}{V}$$

where K_d_ = q_e_/C_e_, m is the adsorbent mass and V is the solution volume. By substituting the batch mass-balance expression q_e_ = (C_0_ − C_e_)V/m into Equation (8), the K_c_ can be rewritten as Equation (9).

(9) $$K_{C}=\frac{C_{0}-C_{e}}{C_{e}}$$

This expression has been used in previous adsorption thermodynamic studies [79,80] and provides a dimensionless equilibrium term suitable for Van’t Hoff analysis.

The temperature dependence of K_c_ was described by the Van’t Hoff equation (Equation (10)):

(10) $$ln\;K_{C}=\frac{\Delta S^{\circ }}{R}-\frac{\Delta H^{\circ }}{RT}$$

A plot of ln K_c_ versus 1/T was used to obtain ∆H° and ∆S° from the slope and intercept, respectively. The ∆G° at each temperature was calculated using Equation (11).

(11) $$\Delta G^{\circ }=-RTln\;K_{C}$$

where R is the universal gas constant (8.314 J/mol·K) and T is the absolute temperature (K).

### 3.9. Regeneration and Reuse Experiments

Regeneration and reuse experiments were conducted in duplicate over five consecutive adsorption–desorption cycles under the selected batch conditions using 150 mg of Fe_3_O_4_@eggshell NC and 50 mL of a 50 mg/L Cr(VI) solution, with a contact time of 60 min. After each adsorption step, the nanocomposite was magnetically separated from the supernatant using a neodymium magnet. The liquid phase was then withdrawn, and an aliquot was filtered through a 0.22 μm PVDF syringe filter for Cr(VI) determination. Desorption was subsequently performed in the same flask by directly adding the regenerating solution to the recovered adsorbent. Two desorbing media, 0.1 M NaOH and 0.5 M NaCl, were evaluated. In each cycle, the adsorbent was contacted with the regenerating solution for 30 min, followed by three rinsing steps with deionized water (10 min per rinse), and then directly reused in the subsequent adsorption run. The capacity retention (CR) after each reuse cycle was calculated according to Equation (12), where q_e,n_ and q_e,1_ are the adsorption capacities at cycle n and at the first cycle, respectively.

(12) $$CR(\%)=\frac{q_{e,n}}{q_{e,1}}\times 100$$

## 4. Conclusions

A biogenic Fe_3_O_4_@eggshell NC was successfully prepared from *Passiflora ligularis* peel extract and eggshell waste. The selected extract provided a suitable biogenic medium for synthesis, while characterization confirmed the formation of a hybrid nanoparticulate material with preserved magnetite and calcite crystalline phases, oxygen-containing surface functional groups, mesoporosity, and superparamagnetic behavior with a minor blocked contribution.

Cr(VI) removal was favored under moderately acidic conditions, and the combined kinetic and equilibrium analyses supported a heterogeneous uptake process with progressive saturation. In particular, the pseudo-first-order model best described the adsorption kinetics, whereas the Sips model provided a useful interpretation of equilibrium behavior, indicating surface heterogeneity and progressive saturation at higher concentrations. Thermodynamic analysis at 50 mg/L confirmed the exothermic nature of the process and showed that adsorption was favored up to 308 K, while reuse tests indicated moderate reusability during the first three cycles, with better performance under alkaline regeneration. Overall, these results show that Fe_3_O_4_@eggshell NC is a waste-derived hybrid material that combines controlled green synthesis, magnetic recoverability, and favorable Cr(VI) adsorption behavior. Further studies should include techno-economic analysis and life-cycle assessment (LCA) to support scale-up and assess the sustainability profile of the proposed material.

## Figures and Tables

**Table 1:** Experimental matrix and DPPH inhibition results for the 2^3^ factorial design used for *Passiflora ligularis* extract preparation.

Run	Ethanol (%)	Temperature (°C)	Time (min)	DPPH Inhibition (%)
E1	60	50	60	57.10 ± 0.62
E2	90	50	60	49.83 ± 2.23
E3	60	70	60	38.86 ± 1.15
E4	90	70	60	22.42 ± 0.49
E5	60	50	90	33.35 ± 1.70
E6	90	50	90	32.74 ± 1.70
E7	60	70	90	37.57 ± 2.19
E8	90	70	90	32.33 ± 1.81
CP	75	60	75	48.81 ± 1.53

**Table 2:** Specific surface area of functionalized Fe_3_O_4_@eggshell NC.

Condition	Surface Area (m^2^/g)
M1	142.71
M2	111.17
M3	155.88
M4	103.18
CP1	127.15
CP2	112.98
CP3	127.18

**Table 3:** Kinetic model parameters for Cr(VI) adsorption by Fe_3_O_4_@eggshell NC.

Model	Parameters	Values
PFO	q_e_ (mg/g)	22.2701
	k_1_ (min^−1^)	0.2331
	R^2^	0.9982
	χ^2 red^	0.1117
PSO	q_e_ (mg/g)	23.1163
	k_2_ (g/(mg min))	0.0213
	R^2^	0.9851
	χ^2 red^	0.9395

**Table 4:** Intraparticle diffusion parameters for Cr(VI) adsorption by Fe_3_O_4_@eggshell NC.

Region	k_id_ (mg/(g min^1/2^))	C (mg/g)	R^2^
I	6.5485	0.0924	0.9993
II	0.7098	18.4678	0.8933
III	−0.0587	22.7907	0.2834

**Table 5:** Fitted isotherm parameters for Cr(VI) adsorption on Fe_3_O_4_@eggshell NC.

Models	Parameters	Values
Langmuir	q_max_ (mg/g)	74.0599
	K_L_ (L/mg)	0.0123
	R^2^	0.9783
	χ^2^	3.0714
Freundlich	K_F_ (mg/g) (L/mg)^1/n^	1.4147
	n	1.3059
	1/n	0.7660
	R^2^	0.9701
	χ^2^	4.2444
Sips	q_s_ (mg/g)	50.8142
	K_s_ (L/mg)	0.0241
	n_s_	1.1960
	R^2^	0.9803
	χ^2^	3.7286
Temkin	B_T_ (mg/g)	11.7505
	A_T_ (L/mg)	0.2247
	R^2^	0.9753
	χ^2^	3.3540

**Table 6:** Standard thermodynamic parameters for Cr(VI) adsorption on Fe_3_O_4_@eggshell NC at C_0_ = 50 mg/L.

Temperature (K)	ΔG° (kJ/mol)	ΔH° (kJ/mol)	ΔS° (J/mol·K)
293	−1.368		
298	−1.109		
308	−0.489	−16.792	−52.813
318	0.079		
328	0.212		

**Table 7:** Magnetic adsorbents reported for Cr(VI) removal: experimental conditions and performance parameters.

Adsorbent and Composition	pH	Dosage (mg/mL)	C_0_ (mg/L)	Time (min)	Removal (%)	Reported Isotherm Model	Reference
MES: Fe_3_O_4_-magnetized eggshell	2.0	12.5	20–30	30	95.00	Langmuir	[23]
EM: Calcined eggshell coated with Fe_3_O_4_ nanoparticles	6.0	1.5	120	60	94.60	Freundlich	[57]
ESM (2:1): Eggshell–magnetite biocomposite synthesized with ethylene glycol	3.0	1.0	12.84	15	99.05	Freundlich	[24]
QBC/MNPs: Quinoa Biochar modified with Fe_3_O_4_ nanoparticles	4.0	2.0	25–200	60	93.62	Freundlich	[59]
Fe_3_O_4_@eggshell: green nanocomposite prepared from *Passiflora ligularis* extract and eggshell-derived matrix	4.0	3.0	50	60	75.98	Sips	This work

**Table 8:** Non-linear isotherm models used for equilibrium fitting.

Model	Non-Linear Equation	Parameters	Meaning (Units)
Langmuir	$q_{e}=\frac{q_{m}K_{L}C_{e}}{1+K_{L}C_{e}}$	q_m_, K_L_	q_m_: maximum adsorption capacity (mg/g), K_L_: Langmuir constant (L/mg)
Freundlich	$q_{e}=K_{F}C_{e}^{1/n}$	K_F_, n	K_F_: Freundlich constant (mg/g) (L/mg)^1/n^, n: heterogeneity factor
Sips	$q_{e}=\frac{q_{s}(K_{s}C_{e}{)}^{n_{s}}}{1+(K_{s}C_{e}{)}^{n_{s}}}$	q_s_, K_s_, n_s_	q_s_: Sips maximum adsorption capacity (mg/g); K_s_: Sips constant (L/mg); n_s_: heterogeneity parameter
Temkin	qe=BT ln ATCe	B_T_, A_T_	B_T_: Temkin constant (mg/g), A_T_: Temkin equilibrium binding constant (L/mg)

## Data Availability

The authors declare that the data supporting the findings of this study are available within the paper and its Supplementary Information Files. Should any raw data files be needed in another format, they are available from the corresponding author upon reasonable request.

## Supplementary Materials

The following supporting information can be downloaded at: https://www.mdpi.com/article/10.3390/molecules31183159/s1, Table S1: Independent variables used in the 2^3^ factorial extraction design; Table S2: Experimental matrix for the 2^2^ factorial design for the Fe_3_O_4_@eggshell NC synthesis; Table S3: ANOVA summary for BET specific surface area (SSA): effects of pH, eggshell mass, and their interaction (central points included); Table S4: Semi-quantitative EDS elemental composition (wt.%) of eggshell powder, green-synthesized Fe_3_O_4_ NPs, and the Fe_3_O_4_@eggshell NC; Table S5: Magnetic parameters (saturation magnetization, M_S_; remanent magnetization, M_R_; and coercive field, H_C_) of the green-synthesized Fe_3_O_4_ NPs and the Fe_3_O_4_@eggshell NC; Table S6: Capacity retention of the Fe_3_O_4_@eggshell NC over five consecutive adsorption–regeneration cycles, calculated relative to the first cycle; Figure S1: SEM micrograph and EDS spectrum of the eggshell powder; Figure S2: SEM micrograph and EDS spectrum of the green-synthesized Fe_3_O_4_ NPs; Figure S3: Zeta potential of green Fe_3_O_4_ NPs and Fe_3_O_4_@eggshell NC. Error bars indicate the standard deviation of three measurements (n = 3); Figure S4: TGA curves of (a) eggshell powder, (b) green Fe_3_O_4_ NPs, and (c) Fe_3_O_4_@eggshell NC showing thermal transitions associated with moisture loss, organic decomposition, and magnetic phase stability; Figure S5: Van’t Hoff plot for Cr(VI) adsorption on Fe_3_O_4_@eggshell NC at an initial Cr(VI) concentration of 50 mg/L. Error bars indicate the standard deviation of three measurements (n = 3); Figure S6: Cr(VI) removal efficiency over five consecutive adsorption–regeneration cycles using (a) 0.1 M NaOH and (b) 0.5 M NaCl as regenerating agents. Error bars indicate the standard deviation of three measurements (n = 3).

## Abbreviations

The following abbreviations are used in this manuscript:

ABTS	2,2′-Azinobis(3-ethylbenzothiazoline-6-sulfonic acid)
ANOVA	Analysis of variance
ATR	Attenuated total reflectance
BET	Brunauer–Emmett–Teller
BJH	Barrett–Joyner–Halenda
COD	Crystallography Open Database
CP	Central point
CR	Capacity retention
DNA	Deoxyribonucleic acid
DPC	1,5-Diphenylcarbazide
DPPH	2,2-Diphenyl-1-picrylhydrazyl
EDS	Energy-dispersive X-ray spectroscopy
EDX	Energy-dispersive X-ray spectroscopy
EM	Calcined eggshell coated with Fe_3_O_4_ nanoparticles
ESM	Eggshell–magnetite biocomposite synthesized with ethylene glycol
FC	Field-cooled
FE-SEM	Field-emission scanning electron microscopy
FIB-SEM	Focused ion beam–scanning electron microscopy
FTIR	Fourier-transform infrared spectroscopy
HC	Coercivity
IEP	Isoelectric point
LCA	Life-cycle assessment
LOD	Limit of detection
LOQ	Limit of quantification
MES	Fe_3_O_4_ magnetized eggshell
MNPs	Magnetic nanoparticles
MR	Remanent magnetization
MS	Saturation magnetization
NC	Nanocomposite
NPs	Nanoparticles
PFO	Pseudo-first-order
PSO	Pseudo-second-order
PVDF	Polyvinylidene fluoride
QBC	Quinoa biochar
SEM	Scanning electron microscopy
TEM	Transmission electron microscopy
TGA	Thermogravimetric analysis
UV–Vis	Ultraviolet–visible
VSM	Vibrating sample magnetometry
XRD	X-ray diffraction
ZFC	Zero-field-cooled
