# Supplementary material for: Biogenic Fe3O4@eggshell Nanocomposite: Synthesis, Physicochemical Properties, and Cr(VI) Removal in Aqueous Media
Source: Molecules. 2026 Sep 8;31(18):3159. doi: 10.3390/molecules31183159 (PMC13609887; doi:10.3390/molecules31183159)
Supplement: Supplementary file 1 [file molecules-31-03159-s001.zip › molecules-4502470-supplementary.pdf]

**Supplementary information**

**"Biogenic $Fe_3O_4$@eggshell Nanocomposite: Synthesis, Physicochemical Properties, and Cr(VI) Removal in Aqueous Media"**

**Tables**

**Table S1.** Independent variables used in the $2^3$ factorial extraction design.

| Factor | Low (−1) | High (+1) |
|---|---|---|
| Solvent concentration (%) (A) | 60 | 90 |
| Extraction temperature (°C) (B) | 50 | 70 |
| Extraction time (min) (C) | 60 | 90 |

**Table S2.** Experimental matrix for the $2^2$ factorial design for the $Fe_3O_4$@eggshell NC synthesis.

| Condition | pH | Eggshell (g) |
|---|---|---|
| M1 | 10 | 1.0 |
| M2 | 10 | 2.0 |
| M3 | 12 | 1.0 |
| M4 | 12 | 2.0 |
| CP1 | 11 | 1.5 |
| CP2 | 11 | 1.5 |
| CP3 | 11 | 1.5 |

**Table S3.** ANOVA summary for BET specific surface area (SSA): effects of pH, eggshell mass, and their interaction (central points included)

| Source | Df | Sum Sq | Mean Sq | F value | Pr(>F) |
|---|---|---|---|---|---|
| pH | 1 | 6.7 | 6.7 | 0.100 | 0.7820 |
| Eggshell | 1 | 1774.6 | 1774.6 | 26.468 | 0.0358* |
| Iscubic (design) | 1 | 57.7 | 57.7 | 0.860 | 0.4515 |
| pH × Eggshell | 1 | 111.9 | 111.9 | 1.669 | 0.3256 |
| Residuals | 2 | 134.1 | 67.0 | — | — |

* Significant at $p < 0.05$

**Table S4**. Semi-quantitative EDS elemental composition (wt.%) of eggshell powder, green-synthesized $Fe_3O_4$ NPs and the $Fe_3O_4$@eggshell NC

| Element | Eggshell (%) | $Fe_3O_4$ NPs (%) | $Fe_3O_4$@eggshell NC(%) |
|---|---|---|---|
| O | 50.0 ± 0.02 | 50.1 ± 2.3 | 63.3 ± 3.9 |
| Ca | 49.6 ± 0.2 | – | 7.8 ± 4.0 |
| Fe | – | 41.0 ± 2.3 | 15.5 ± 5.8 |
| C | Not detected | 7.3 ± 0.6 | 13.4 ± 2.2 |
| Na | Trace (<0.1) | 1.0 ± 0.1 | - |
| Mg | 0.4 ± 0.1 | Trace (<0.5) | ≈ 0.1 |
| Al, Si, P | – | Trace (<1) | - |

**Table S5**. Magnetic parameters (saturation magnetization, $M_S$; remanent magnetization, $M_R$; and coercive field, $H_C$) of the green-synthesized $Fe_3O_4$ NPs and the $Fe_3O_4$@eggshell NC

| Sample | $M_S$ (emu/g) | $M_R$ (emu/g) | $H_C$ (Oe) |
|---|---|---|---|
| Green $Fe_3O_4$ NPs | 46.4 ± 0.3 | 0.18± 0.05 | 1.9 ± 0.5 |
| $Fe_3O_4$@ggshell NC | 8.7 ± 0.1 | 0.038 ± 0.001 | 1.8 ± 0.5 |

**Table S6**. Capacity retention of $Fe_3O_4$@eggshell NC over five consecutive adsorption–regeneration cycles, calculated relative to the first cycle

| Cycle | NaOH 0.1 M (%) | NaCl 0.5 M (%) |
|---|---|---|
| 1 | 100.00 | 100.00 |
| 2 | 97.15 | 84.67 |
| 3 | 95.94 | 76.06 |
| 4 | 65.20 | 56.88 |
| 5 | 67.60 | 51.66 |

## Figures

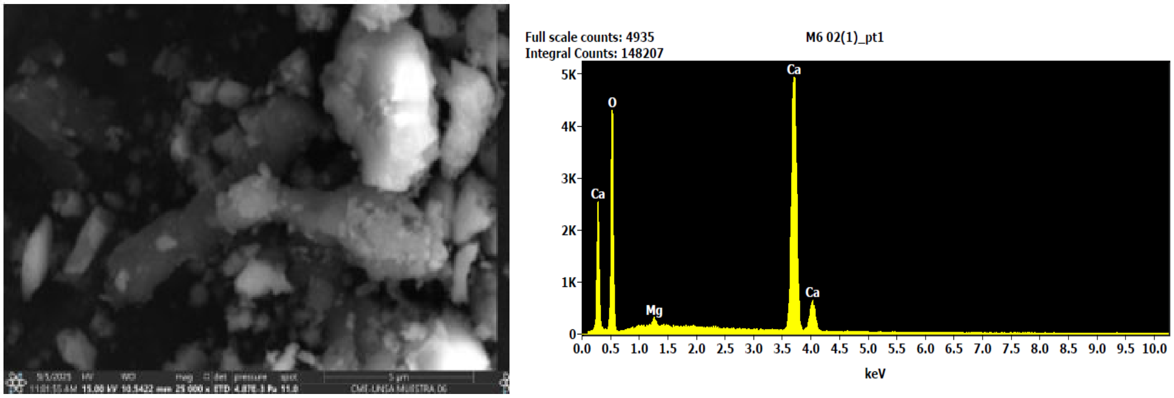


(a) (b)

**Figure S1.** (a) SEM micrograph and (b) EDS spectrum of the eggshell powder.

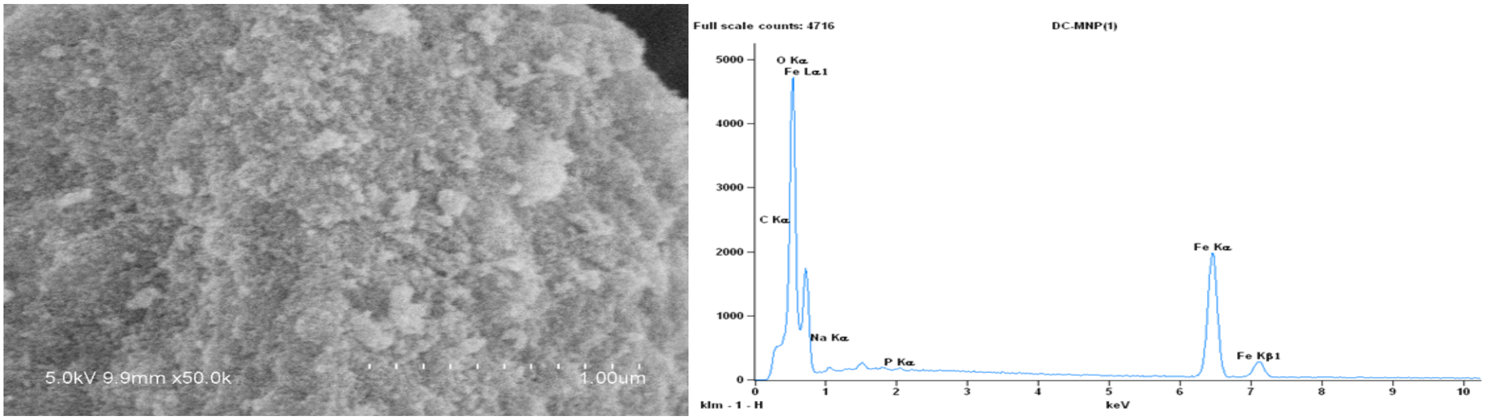


(a) (b)

**Figure S2.** (a) SEM micrograph and (b) EDS spectrum of the green-synthesized $Fe_3O_4$ NPs

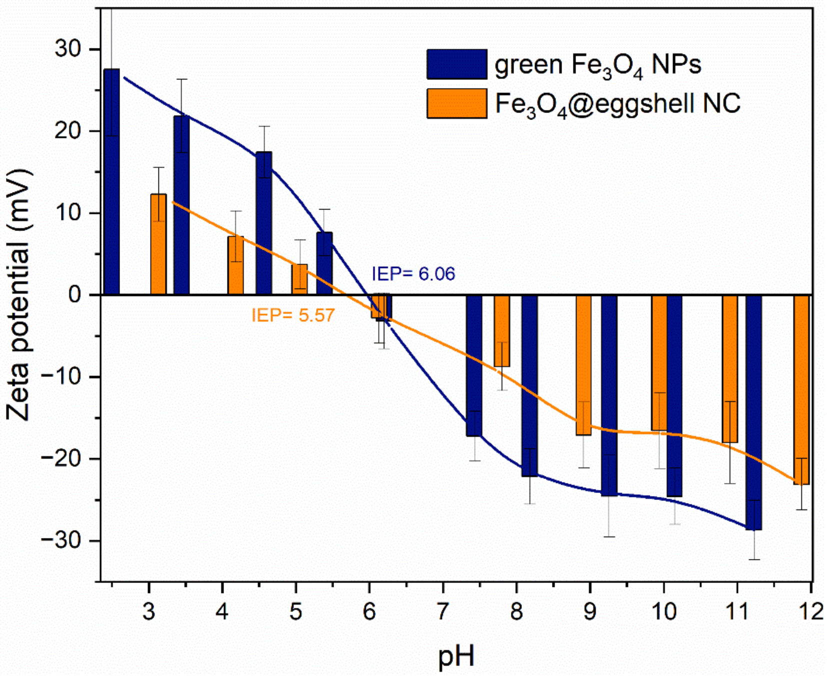


**Figure S3.** Zeta potential of green $Fe_3O_4$ NPs and $Fe_3O_4$@eggshell NC. Error bars represent the standard deviation of three measurements (n = 3).

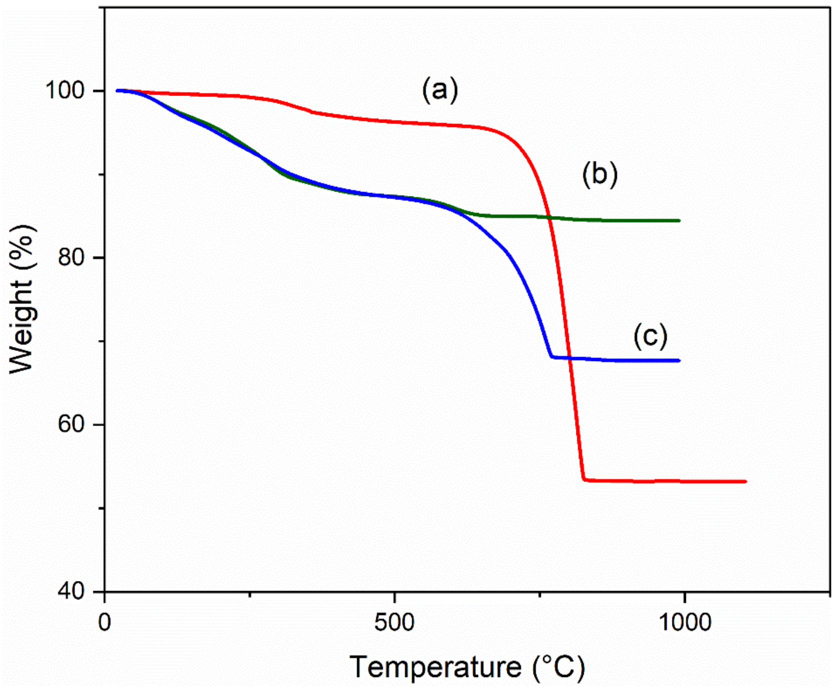


**Figure S4.** TGA curves of (a) eggshell powder, (b) green $Fe_3O_4$ NPs, and (c) $Fe_3O_4$@eggshell NC, showing thermal transitions associated with moisture loss, organic decomposition, and magnetic phase stability

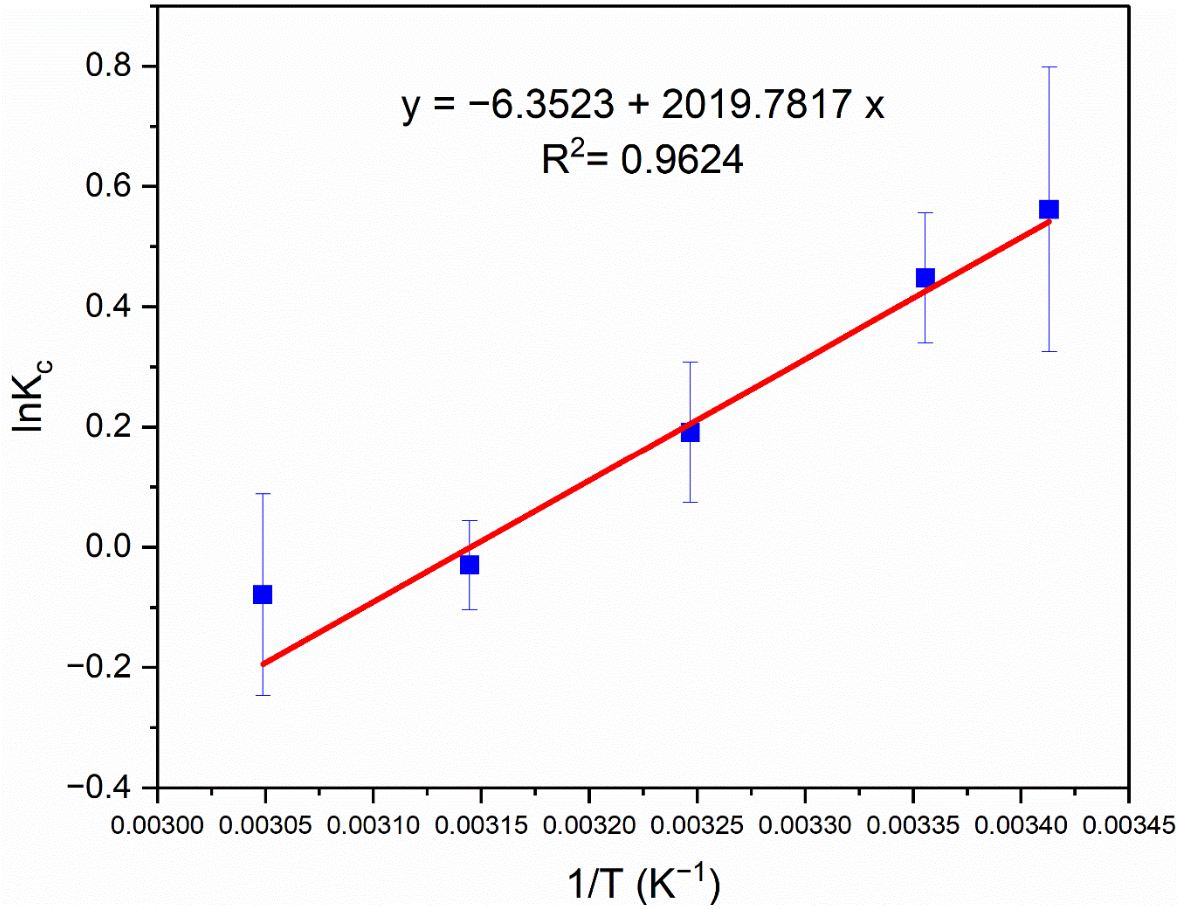


**Figure S5.** Van't Hoff plot for Cr(VI) adsorption on $Fe_3O_4$@eggshell NC at an initial Cr(VI) concentration of 50 mg/L. Error bars represent the standard deviation of three measurements (n = 3).

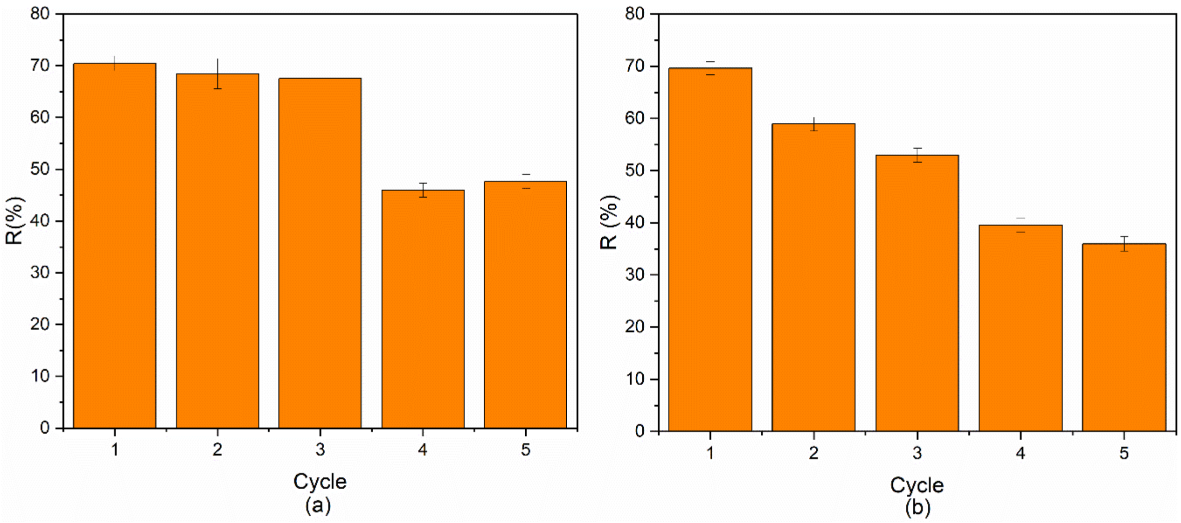


**Figure S6**. Cr(VI) removal efficiency over five consecutive adsorption–regeneration cycles using (a) 0.1 M NaOH and (b) 0.5 M NaCl as regenerating agents. Error bars represent the standard deviation of three measurements (n = 3).
